# Determinants and outcomes of eHealth literacy in healthy adults: A systematic review

**DOI:** 10.1371/journal.pone.0291229

**Published:** 2023-10-04

**Authors:** Ariesta Milanti, Dorothy Ngo Sheung Chan, Anselmus Aristo Parut, Winnie Kwok Wei So

**Affiliations:** 1 The Nethersole School of Nursing, Faculty of Medicine, The Chinese University of Hong Kong, Hong Kong SAR, Hong Kong, China; 2 Independent Scholar, Labuan Bajo, Flores, Indonesia; Xiamen University - Malaysia Campus: Xiamen University - Malaysia, MALAYSIA

## Abstract

**Background:**

eHealth has shown many benefits in health promotion and disease prevention. For engaging in and taking advantage of eHealth, eHealth literacy is essential. This systematic review aims to summarise and examine the existing evidence on determinants and outcomes of eHealth literacy in healthy adults.

**Methods:**

We searched the relevant peer-reviewed articles published in English in six databases: MEDLINE, EMBASE, PsycINFO, CINAHL, Cochrane Library and ProQuest. The inclusion criteria of the review were: 1) studies examining ‘eHealth literacy’, which refers to the ability to search, select, judge and apply online health information to address or solve health problems and to improve wellbeing; 2) the type of study included observational and experimental studies, mixed method studies or qualitative studies; 3) the participants were healthy adults; 4) the main outcomes were the determinants (i.e. influencing or associated factors) and outcomes (i.e. benefits and disadvantages) of eHealth literacy. Articles were assessed by two reviewers using the Joanna Briggs Institute critical appraisal tool. A conceptual model to map the determinants and outcomes of eHealth literacy in healthy adults into the non-modifiable, individual, social and community networks and structural layers was developed to classify the identified determinants and outcomes.

**Results:**

Forty-four studies were included in this review, of which 43 studies were cross-sectional and one was qualitative. eHealth literacy determinants included age, sex, literacy factors, socioeconomic factors and language. eHealth literacy outcomes included better general health promotion behavior, COVID-19 preventive behaviors, psychological wellbeing, social support, self-rated health and health service utilisation.

**Conclusions:**

Our results showed that eHealth literacy has multi-layered determinants and positive outcomes. Different strategies at different policy levels are needed to improve the eHealth literacy levels of healthy adults.

## Introduction

Advances in information and communication technologies (ICT) have benefitted services in all sectors including health. The use of ICT for supporting health and health-related fields, including health care services, health surveillance, health literature and health education can be referred to as electronic health (eHealth) [1]. eHealth is currently recognised as one of the fastest growing areas in today’s health field [2]. The World Health Organisation also acknowledged the potential of eHealth to achieve the Sustainable Development Goals, especially in supporting health promotion and disease prevention in all countries, by enhancing the quality, accessibility and affordability of health services [3]. This can be achieved by various means of eHealth including telehealth, eLearning, mHealth and social media [4].

In order to engage in, and take the advantage of eHealth, individuals need eHealth literacy [4]. eHealth literacy may be defined as “one’s ability to seek, find, understand, appraise and apply the health information from electronic sources to address or solve a health problem” [5]. eHealth literacy is a complex entity. The challenge of identifying, appraising and understanding health information from online sources is already complex, especially when the information is to be used to make health-related decisions or to inform health behavioral changes [6]. eHealth literacy is also dynamic; it is a process-oriented skill that evolves over time [5]. Given ICT’s rapid development and changes in personal, social and environmental contexts, individuals must continue to develop their eHealth literacy for their own empowerment, in order to achieve health-related outcomes [5, 7].

Evidence indicates that eHealth literacy is associated with health outcomes, such as health promoting behaviors and facilitating psychological well-being across various population groups [6, 8, 9]. On the other hand, according to Norman and Skinner [5], eHealth literacy can be associated with individual’s educational background, health status and presenting health issue when encountering the eHealth resources, motivation for seeking the information and the technology used. Apart from these hypotheses, empirical studies have found many other socio-demographic and literacy variables as important determinants of eHealth literacy [10–14].

Previous systematic reviews related to eHealth literacy were carried out across different populations, including college students [15], older adults [16, 17], older adults in China [8], people living with HIV [18], and underserved population in the United States [19]. Previous systematic reviews have also addressed eHealth literacy interventions [17, 20, 21], eHealth literacy tools [22, 23], health literacy in eHealth era [24], eHealth definition [25], policy issues in eHealth [26], and eHealth literacy’s relationships with health-related behaviors [27]. However, to the best of our knowledge, there is still no systematic review to summarise the evidence of eHealth literacy’s determinants and outcomes, unrelated to a specific intervention. To address this perceived gap, this systematic review aims to summarise and appraise the existing research which has examined the determinants and outcomes of eHealth literacy in healthy adults. Understanding the determinants and outcomes of eHealth literacy can provide valuable insights into the factors that influence individuals’ ability to use technology to access, understand, and use health information, and the impact that eHealth literacy has on health outcomes. This information can then be used to develop interventions and policies to promote eHealth literacy and improve health outcomes in healthy adults. Focusing on the adult population is important since this population makes up the largest age group in the world [28]. Moreover, by focusing the study population on healthy adults, the researchers can minimise the potential confounding effects of health conditions or other factors that may influence eHealth literacy.

## Methods

The protocol for this review was registered in PROSPERO International Prospective Register of Systematic Reviews (reference number: CRD42021271346).

### Search strategy

We searched six electronic databases: MEDLINE, EMBASE, PsycINFO, CINAHL, Cochrane Library and ProQuest to identify relevant studies. Relevant grey literature including dissertations and theses were identified from ProQuest. These databases were selected since they are among the largest and most widely recognized as authoritative sources in the fields of healthcare, medical, nursing, psychology, and behavioral sciences. They offer a comprehensive coverage of literature relevant to the eHealth literacy topic. Including other databases might introduce redundancy in the review process. These databases were chosen to strike a balance between comprehensive coverage and efficiency.

A search strategy was developed to capture the key elements of the topic of interest, i.e.: determinants and outcomes of eHealth literacy. Keywords were combined using AND and OR Boolean operators. The year of publication was not limited. The detailed search strategy is presented in S1 Table.

### Selection process

Studies were included if they met these criteria: 1) studies examining ‘eHealth literacy’; 2) the type of study included observational and interventional studies, mixed methods studies, and qualitative studies; 3) population/participants were healthy adults (defined as individuals aged over 18–64 years [29] without significant chronic health conditions or disabilities); 4) the main outcomes were the determinants (i.e. influencing or associated factors) and outcomes (i.e. positive and negative outcomes) of eHealth literacy. Intervention and comparator were beyond the scope of this review. Reviews, editorials, expert’s opinion, letters and conference papers were excluded. Articles written in non-English languages were also excluded.

All citations were managed using EndNote 20 software (Clarivate Analytics, PA, USA). Duplicates were identified and removed electronically (using ’Find Duplicates’ feature in EndNote) and manually. After removing duplicates, the titles and abstracts of the records were screened against the inclusion and exclusion criteria by two independent reviewers (AM and AAP), using EndNote 20 software. The records which clearly did not meet the inclusion criteria were excluded from the review. Full texts of all retained articles were also screened independently by two reviewers (AM and AAP). Disagreement was resolved by discussion between the two reviewers. The selection process and reasons for exclusion were documented using a PRISMA flow diagram.

### Assessment of methodological quality

AM and AAP independently carried out the critical appraisal for each study. The Joanna Briggs Institute (JBI) critical appraisal tools were used, according to each study’s design, to assist in examining the trustworthiness and risk of bias of the included studies [30]. The JBI critical appraisal tools are widely recognized within the field of evidence synthesis for their comprehensive approach to evaluating the risk of bias based on the study’s design, conduct, and analysis. While other tools, such as the Cochrane Risk of Bias Tool, Newcastle-Ottawa Scale (NOS), Appraisal tool for Cross-Sectional Studies (AXIS), and A measurement Tool to Assess Systematic Reviews (AMSTAR 2), mostly focus on specific study types and their respective attributes, JBI tools offer a broader approach in comprehensively assessing various study designs. Therefore, the JBI tools were deemed suitable for systematically reviewing the determinants and outcomes of eHealth literacy across different research methodologies.

### Data collection process

AM extracted the data using a standardised extraction sheet in Microsoft Word to record the study characteristics of included articles. Afterwards, AAP checked and amended the data extraction results as required.

### Data items

The information included: a) the details of the study’s aim, b) design, c) sample size, d) types of participants, e) participants’ characteristics, f) eHealth literacy instrument and g) mean score of eHealth literacy. In addition, the determinants and outcomes were also extracted from the included studies.

### Data synthesis

A conceptual model illustrating the determinants and outcomes of eHealth literacy was developed, based on Dahlgren and Whitehead’s Determinants of Health Model [31]. According to Dahlgren and Whitehead [31], there are a series of layers which influence health; every layer has its own implication for policy making. The outermost layer is the major structural environment which covers the cultural and environmental conditions. Under the major structural environment, there are the living and working conditions which are determined by various factors including education, employment and health care. Next, there are the social and community networks where an individual can have mutual support from family, friends and the local community. The inner layer after social support involves individual factors; for example, eating and smoking. Finally, the innermost layer contains the non-modifiable factors over which we have little or no control, including age and sex [31]. Data from the included studies were synthesised and applied to this model by extracting the information on the determinants and outcomes of eHealth literacy and mapping the findings onto the layers of Dahlgren and Whitehead’s model. Finally, areas where interventions or policies can be targeted related to each determinant were identified.

## Results

### Study selection

**Fig 1:**
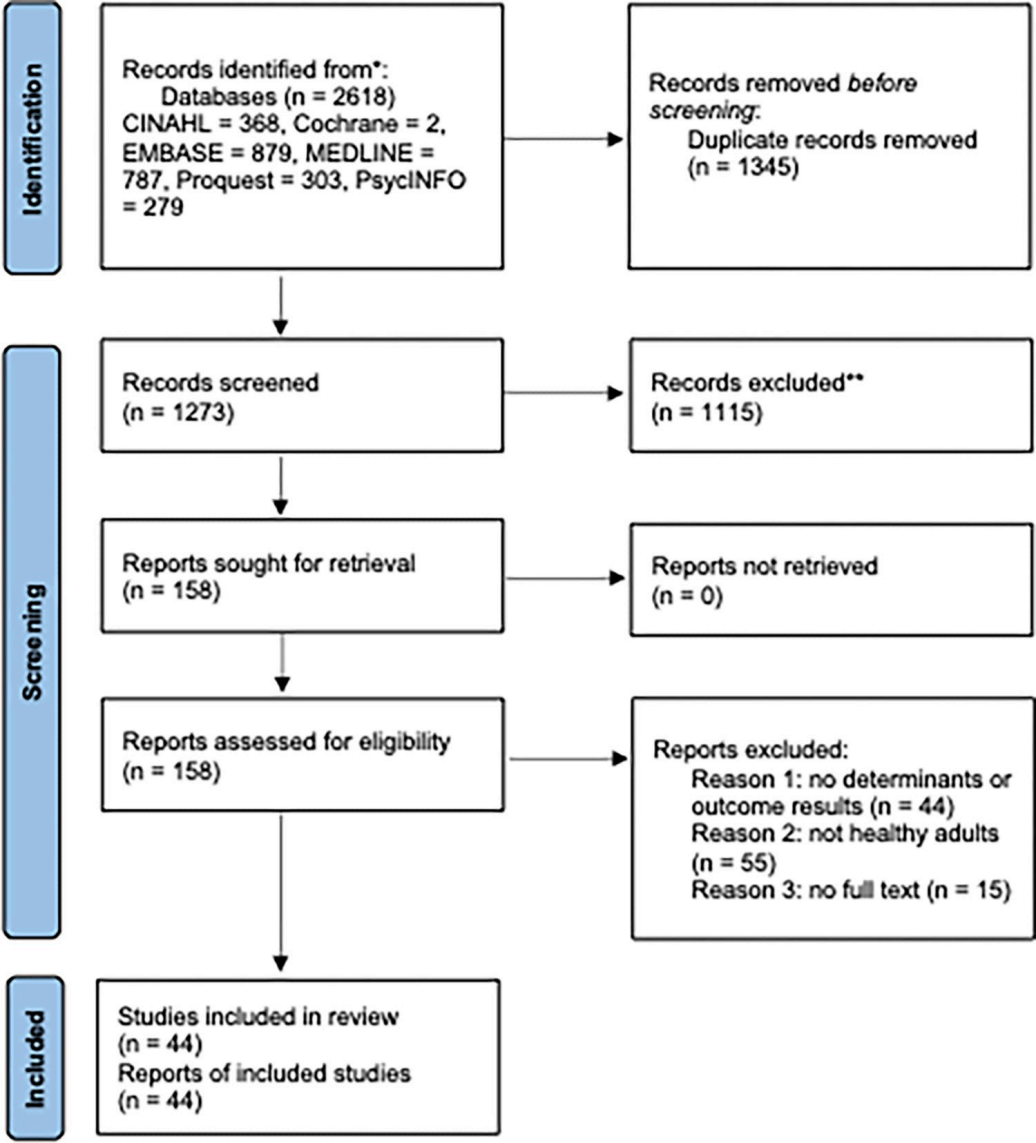
PRISMA flowchart of study selection process.

Forty-four studies were eligible for inclusion (Fig 1). The studies were from the US (n = 9), China (n = 6), South Korea (n = 5), Taiwan (n = 4), Japan (n = 3), Israel (n = 2), Ethiopia (n = 2), Pakistan (n = 2), Hong Kong (n = 2), Greece (n = 2), Cyprus and Greece (n = 1), Singapore (n = 1), Ghana (n = 1), Vietnam (n = 1), Denmark (n = 1), Turkey (n = 1), and the UK (n = 1).

This review included 43 cross-sectional studies and one qualitative study (S1 Table). As the causal relationship could not be drawn from the cross-sectional study design, the determinants and outcomes were putative only.

Eighteen (41.0%) studies recruited lay people, mostly internet users [10, 32–48]. Eighteen (41.0%) studies involved university students as their participants [9, 49–65]. Five (11.3%) studies had health care professionals as the participants [66–70]. Two (4.5%) studies recruited carers of children with special health care needs [11, 12]. Lastly, one (2.2%) study involved carers of people with dementia [71].

Thirty four (77.2%) studies [10, 32‒39, 41–48, 50–53, 56, 62–64] used Norman and Skinner’s eHealth literacy scale (eHEALS) [72]. Four (9.1%) studies [54, 55, 61, 65] used the Chiang et al. eHealth literacy scale (eHLS) [73]. Two (4.5%) studies [9, 57] used Van Der Vaart and Drossaert’s Digital Health Literacy Instrument (DHLI). Two (4.5%) studies [40, 60] used eHealth Literacy Questionnaire (eHLQ) developed by Norgaard et al. (2015) [74]. One (2.2%) study used the European Commission’s digital competency framework [70]. One study (2.2%) [49] was a qualitative study and did not use any eHealth literacy measuring instrument.

### Quality assessment in studies

The quality assessment, using the JBI instrument for each included study, is available in the Supplementary materials. We quantified the number of the critical appraisal components which were fulfilled in each study. The majority of the quantitative studies (n = 24) had 6 out of 8 components fulfilled. Most of the unfulfilled criteria were about identification of confounding factors and strategies to address them. Meanwhile, the qualitative study (n = 1) had 7 out of 10 components fulfilled. This qualitative study did not state the cultural or theoretical location of the researcher, the influence of the researcher on the research, and the ethical considerations of the study. None of the papers was excluded based on the quality evaluation.

### Determinants of eHealth literacy

**Fig 2:**
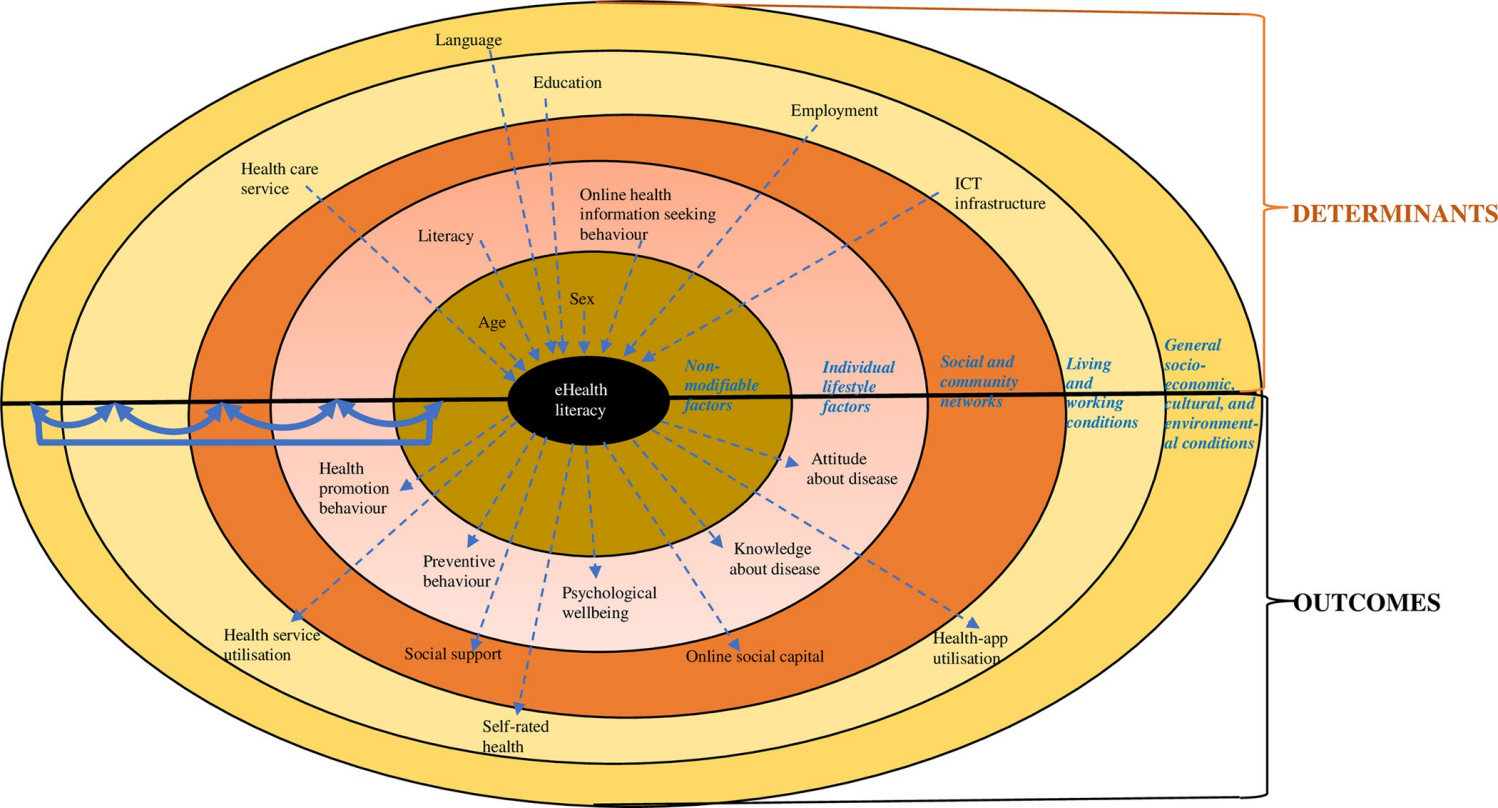
Conceptual model of eHealth literacy determinants and outcomes.

Summary of findings from studies which address the determinants of eHealth literacy are presented in Table 1 and is illustrated in Fig 2.

**Table 1:** Determinants of eHealth literacy.

Category	Sub-category	Determinant	References
Non-modifiable factors	Age	Younger age	[10–12, 32, 39, 69]
		Older age	[46, 62]
	Sex	Male	[32, 37, 46, 67]
		Female	[57]
Individual factors	Literacy	Health literacy	[71]
		Internet literacy	[32, 46]
		Computer literacy	[39]
		Information literacy	[39]
		Digital literacy	[62, 70]
	Online health information seeking behavior	Online health information seeking behavior	[52, 63]
		Online health information searching experience	[47]
		Health information orientation	[46]
		Frequently sought health information	[54]
		Internet usage period of over 15 years	[62]
		Intensity of Instagram use	[50]
		Effort in operating ICT devices	[47]
		Satisfaction with information	[57]
		Importance of information	[57]
		Higher perceived trust in online information	[37]
Living and working conditions	Education	Higher education	[10, 11, 39, 41, 46, 47, 60, 70]
		Higher academic level/e.g. year 1, 2, 3	[62]
		Doing medical major	[54]
	Employment	Higher income	[41, 46, 70]
		Government employee	[70]
		Physician	[69]
		Years of experience < 5 years	[69]
		Collegial nurse-physician relationships	[68]
		Nurse participation in hospital affairs	[68]
	Health care service	A very or fairly easy ability to pay for medication	[67]
		A very or fairly easy ability to pay for doctors	[67]
	ICT infrastructure	Availability and accessibility of ICT resources	[49]
		Simple and concise presentation of health information	[49]
		Categorisation of authentic and unauthentic websites	[49]
General socio-economic, cultural and environmental conditions	Language	Language	[11]

#### Non-modifiable determinants

Age and sex were found to be the non-modifiable determinants of eHealth literacy (Fig 2). Studies found inconsistencies in how these two factors were linked with eHealth literacy. In six studies, younger age was found to be associated with higher eHealth literacy [10–12, 32, 39, 69]. Whereas, in a study conducted among online users in China, older age was found to be related with higher eHealth literacy [46]. Inconsistency was also found with regards to sex. Being male was found to be associated with higher eHealth literacy in four studies [32, 37, 46, 67], while being female was found to be related with higher eHealth literacy in only one study [57].

#### Individual determinants

In the individual layer of eHealth literacy determinant model (Fig 2), there are literacy factors and online health information-seeking behavior factors. The literacy factors included health literacy [71], internet literacy [32, 46], computer literacy [39], information literacy [39] and digital literacy [62, 70]. The online health information seeking behaviors included: online health information seeking behavior [52, 63]; health information orientation i.e. the willingness of people to look for health information [46]; internet usage period of over 15 years [62] and intensity of Instagram use [50]. In addition, the users’ satisfaction with information and the importance of information influenced eHealth literacy. A higher perceived trust in online information also influenced eHealth literacy [37, 57]. Moreover, Magsamen-Conrad et al. found that less effort in operating ICT devices was associated with higher eHealth literacy [47].

#### Determinants related to living and working conditions

For the social and community network layer of determinant model, there was no factor found to be the determinant of eHealth literacy. However, for the next layer (living and working conditions) the factors of: education, employment, living conditions and health care services were identified.

Higher education was consistently associated with higher eHealth literacy, as indicated in eight studies [10, 11, 39, 41, 46, 47, 60, 70]. Still related with education, studies conducted among college students found that the higher the academic level (i.e. year 3 compared to year 1) and the students’ major subject both influenced eHealth literacy [54, 62].

Higher income was consistently related to higher eHealth literacy [41, 46, 70]. Studies conducted among health care professionals in Ethiopia found that being a government employee, being a physician, and having less than five years working experience were found to be associated with higher eHealth literacy [69, 70].

Related to the health care service, personal health care financing was found to be linked with eHealth literacy. The ability to pay for medications and doctors were associated with higher eHealth literacy [67].

Other determinants of eHealth literacy are related to ICT infrastructure. In a qualitative study conducted among college students in Pakistan, Adil et al. identified that the access to ICT resources, categorisation of authentic and unauthentic websites and presentation of eHealth information influenced eHealth literacy [49].

#### General socio-economic, cultural and environmental determinants

Finally, in the outermost layer of the eHealth literacy determinant model, which represents the general socio-economic and cultural environments (Fig 2), there is the factor of language to consider. Knapp et al. in a study about the eHealth literacy of low-income parents whose children have special health care needs, found that non-English speaking parents were more likely to demonstrate lower eHealth literacy levels [11].

### Outcomes of eHealth literacy

A summary of findings from the studies which addressed the outcomes of eHealth literacy is presented in Table 2 and is illustrated in Fig 2.

**Table 2:** Outcomes of eHealth literacy.

Category	Sub-category	Outcomes	References
Individual lifestyle factors	Health promotion behavior	Better general health and future intention to maintain health	[58]
		Health promoting behavior	[44, 45, 63, 64, 66].
		Health responsibility	[66]
		Self-care competence	[63]
		Exercise	[33, 39, 53, 58, 61, 67]
		Better physical activity among nurses working on fixed-day and day-evening shifts	[66]
		Healthy eating	[67]
		A balanced diet	[33, 55, 58, 61]
		Lower unhealthy food intake	[55]
		Regular breakfast	[53]
		Regular eating habit	[55]
		Lower risk of being overweight	[53]
		Sleep	[58]
		Getting vaccinations	[58]
		Maintenance of sexual health	[58]
		A lifestyle free of harmful substances	[58]
		Colorectal cancer screening practice	[48]
	Preventive behavior	Higher adherence to COVID-19 preventive behaviors	[10, 41, 45, 51, 56, 64, 67]
	Knowledge about disease	Higher COVID-19 knowledge	[41]
		Higher Colorectal cancer (CRC) knowledge	[48]
	Attitude about disease	Lower COVID-19 conspiracy beliefs	[41]
		More confidence in finding cancer information	[36]
	Psychological wellbeing	Better Psychological wellbeing	[9, 35]
		Higher self-actualisation	[66]
		Lower depression	[40]
		Lower insomnia	[40]
		Lower PTSD	[40]
		Better Stress management	[66]
		Higher sense of coherence	[57]
Social and community networks	Stable friendship and social support	Stable friendships/ social support	[58]
		Better interpersonal relations	[66]
	Online social capital	Online bridging social capital	[50]
Living and working conditions	Self-rated health	Better perceived health outcomes	[43]
		Self-rated health	[34, 55, 60, 70]
		Lower likelihood of suspected COVID-19 symptoms	[67]
	Health service utilisation	Health service utilisation	[65]
	Health-app utilisation	Health-app use efficacy	[42]
		The extent of health-app use	[42]

#### Individual lifestyle-related outcomes

Many studies included in this review were conducted during the COVID-19 pandemic and the outcomes were also related to COVID-19. Seven studies found that higher eHealth literacy was associated with higher adherence to COVID-19 preventive behaviors [10, 41, 45, 51, 56, 64, 67]. A web-based survey of US adults found that higher eHealth literacy was also related to higher COVID-19 knowledge and lower levels of COVID-19 conspiracy beliefs [41]. In addition, a study among health care professionals in Vietnam found that higher eHealth literacy was also linked to a lower likelihood of having suspected COVID-19 symptoms [67].

In terms of psychological outcomes, two studies indicated that higher eHealth literacy was associated with better psychological wellbeing [9, 35]. Another study conducted among hospital nurses in South Korea showed that higher eHealth literacy was associated with a sense of higher self-actualisation (i.e. an individual’s perception of having a sense of purpose in life and thriving for self-development) and better self-management [66]. The results of a survey conducted in China during the COVID-19 pandemic suggested that higher eHealth literacy was related to lower depression, lower insomnia and lower post-traumatic stress disorder (PTSD) [40]. Additionally, a survey amongst university students in Pakistan found a higher sense of coherence of the COVID-19 situation, which encompassed the senses of comprehensibility, manageability and meaningfulness of the COVID-19, was associated with higher eHealth literacy [57].

Five studies showed that eHealth literacy was related to health promoting behavior [44, 45, 63, 64, 66]. eHealth literacy was also associated with general health and future intention to maintain health, according to a study of college students in the US [58]. eHealth literacy was also linked with the issue of personal health responsibility [66]. Moreover, a study conducted among nursing students in South Korea showed that eHealth literacy was associated with self-care competence [63].

Still related to health promoting behaviors, several studies examined the health promoting behaviors more specifically, including exercise, health eating, sleep, a lifestyle free of harmful substances and maintenance of sexual health. Six studies found that eHealth literacy was associated with exercise [33, 39, 53, 58, 61, 67]. A study in South Korea showed that eHealth literacy was related to better physical activity among nurses working on fixed-day and day-evening shifts, but not the among the nurses working rotating shifts [66]. Furthermore, eHealth literacy was associated with better health behaviors including sleep, getting vaccinations, maintenance of sexual health and a lifestyle free of harmful substances [58].

Four studies suggested that eHealth literacy was linked with a balanced diet [33, 55, 58, 61], while one study suggested that eHealth literacy was linked with higher likelihood of healthy eating [67]. A study in Taiwanese college students also found that eHealth literacy was associated with lower intake of unhealthy food and the maintaining of regular eating habits. A study conducted in Japan showed that college students with higher eHealth literacy were more likely to have regular breakfast and to be at lower risk of being overweight [53].

With regards to colorectal cancer (CRC), a study was conducted among internet users in Japan [48]. This study found that eHealth literacy was associated with CRC knowledge and practice [48].

#### Social and community networks-related outcomes

In the social and community networks layer, stable friendships or social support were found to be related with eHealth literacy [58]. People with higher eHealth literacy were more likely to have better interpersonal relationships, according to a study in South Korea [66]. Moreover, people with higher eHealth literacy was also found to be have better online social capital; that is, the accessibility to ties on an online network, in which the latter promotes group norms and trust [50].

#### Living and working conditions outcomes

In the next layer, regarding living and working conditions, health outcomes and health service/health app utilisation were found to be linked to eHealth literacy. According to four studies, eHealth literacy was associated with self-rated health [34, 55, 60, 70]. A study in Israel found that eHealth literacy was related to better perceived health outcomes [43]. On the other hand, eHealth literacy was also found to be linked with health service utilisation [65], health-app use efficacy and the extent of health-app use [42].

The model suggests that eHealth literacy is shaped by a complex interaction of multi-layered determinants (i.e. non-modifiable factors, individual lifestyle, social and community networks, living and working conditions, and general socio-economic and cultural conditions). As a result, eHealth literacy is also associated with various outcomes.

## Discussion

This review has attempted to consolidate the determinants and outcomes of eHealth literacy in healthy adults. We categorised the determinants and outcomes of the eHealth literacy into layers which could influence health, according to Dahlgren and Whitehead’s model [31]. Dahlgren and Whitehead’s model is a useful resource from which to outline strategic approaches to promote greater equity in health through different policy levels for interventions [31], in this case related to eHealth literacy.

In the first layer of eHealth literacy determinants, we identified age and sex as the non-modifiable factors. There are conflicting findings regarding these two factors, but, overall, most studies in this review support ‘younger age’ and ‘male sex’ to be linked with higher eHealth literacy level. One study’s result, that older age was associated with better eHealth literacy, is possibly because the participants were heavy internet users regardless of age [46]. However, this review did not include studies among older adults, therefore, comparisons between younger and older age participants cannot be made. In terms of sex, it is quite surprising that men were more likely to have a higher level of eHealth literacy than women. Prior studies showed that women used health apps [75] and searched health information online [76, 77] more often than men did. Women were also found to be more eHealth literate than men and perceived online information quality better [78, 79]. More studies are needed to clarify how sex differences determine eHealth literacy.

In the second layer of the eHealth literacy determinants, there are individual factors which comprised literacy issues and online health information seeking behavior issues. According to Norman and Skinner’s Lily Model of eHealth literacy, eHealth literacy is the combination of six core literacies, i.e. traditional literacy, media literacy, information literacy, health literacy, scientific literacy and computer literacy [5]. In this review, three literacies including health literacy, computer literacy and information literacy were found to be the determinants of eHealth literacy. However, the other three literacies in the model: traditional, scientific, and media are hypotheses which still need to be tested and proven. Traditional literacy, which is defined as the basic literacy skills of reading, writing, and understanding written language, is comprised of simple tasks which might be overlooked in eHealth literacy research. Despite being so-called ‘traditional’, such literacy is essential in the era of Web 2.0, when internet platforms have more focus on community building, data sharing and co-production [80]. Traditional literacy can be the relevant ability to determine the relevance of online information, as well as in creating self-generated content. According to Norman [7], eHEALS, which is the most frequently used eHealth literacy tool (as noted in this review), is arguably incomplete when required to measure eHealth literacy for the Web 2.0. Further studies are deemed necessary to update eHEALS to fit its purpose in the Web 2.0 internet era. Subscales to assess communication skills in online social interactions and in synthesising contents are some instances of items that could be developed for updated version of eHEALS. Studies are also needed to assess the traditional literacy, scientific literacy and media literacy. The latter is especially important in combating disinformation and bridging the digital divide [81].

In the living and working conditions layer, there is socioeconomic status. This factor includes education, employment, income and ease of paying for health care, all of which play important roles in determining the eHealth literacy levels of healthy adults. This finding is consistent with the result of a previous systematic review of eHealth literacy in an underserved population in the US [19]. In the countries where there is no universal health coverage, such as the US, those who are poor and uninsured may have lesser access to health care, confirming their status as a vulnerable and underserved population [19]. Economically disadvantaged people may also have decreased opportunity to access technology [19]. Even if they have access to mobile devices and the internet, this population is also more likely to have lower levels of education; the latter being a strong predictor of eHealth literacy, as shown in several studies [10, 11, 41, 47]. Therefore, it is particularly important to assess the population with lower socioeconomic status regarding their eHealth literacy levels, and to intervene where necessary, in order to address the digital divide between this population and the wider population with higher levels of education and therefore of eHealth literacy.

Referring to Dahlgren and Whitehead’s model [31], different policy levels may apply in different layers of the health determinant. Policy level 1 is for the major structural environment, in which the language factor is situated. For addressing this determinant, national-level strategies, such as incorporating language services in the national health care system, are needed [82]. Having national and health industry investments to develop population-based language services by using ICT is also recommended [82]. Furthermore, policy level 2, which aims to improve living and working conditions can achieve that aim by, for example, a) providing the welfare benefits through the social security sector, and improving b) health care services, c) the education system and d) the ICT infrastructure [31]. Policy level 3, aiming at strengthening social and community support is not applicable, as there is no eHealth literacy determinant identified in this layer. Policy level 4 aims at influencing individual lifestyles [31]. The policy can include developing programmes to enhance eHealth literacy skills. Finally, for the innermost layer, i.e. the non-modifiable factor, the intervention can be integrated into the educational improvement of the eHealth literacy for older adults of both sexes and women of all ages.

With regard to the outcomes, eHealth literacy was found to have several positive outcomes. Of note, most eHealth literacy studies within this review were conducted during the COVID-19 pandemic. Indeed, in this pandemic, eHealth literacy became more relevant than ever, since most of the COVID-19 information, including misinformation and disinformation, is disseminated through the internet [10]. eHealth literacy can positively influence COVID-19 preventive behaviors [10, 41, 45, 51, 56, 64, 67], knowledge and attitude about disease [41], and facilitate psychological wellbeing [9, 35]. General health promotion behaviors such as healthy eating, exercise and sleep are also the outcomes of eHealth literacy [44, 45, 63, 64, 66]. In addition, eHealth literacy positively influences social and community networks, as well as living and working conditions [58]. Nonetheless, these are putative outcomes only. Randomised controlled trials are needed to infer the causal relationships between eHealth literacy and these outcome benefits.

A systematic review on the influencing factors of eHealth literacy among Chinese older adults [8] showed several similar findings with our study results, i.e. age, sex, education, socioeconomic status, and language influenced the levels of eHealth literacy [8]. These similarities suggest the factors which may be the universal determinants of eHealth literacy across different culture and population. However, our present study found that eHealth literacy was also influenced by literacy factors and ICT infrastructure. Another difference is that physical and psychological conditions, marital status, and being the family carer were found to be associated with the Chinese older adults’ levels of eHealth literacy [8]. These differences may indicate the factors that may be unique to older adult population, especially in China. With regards to outcomes, similar to our study findings, a recent systematic review concluded that eHealth literacy is associated with health-promoting behaviors including regular eating and exercise, and compliance with disease prevention behaviors [27]. These similar and different findings show the importance of conducting systematic review to find the consistent and inconsistent patterns across different studies.

### Study strengths

The systematic review offers comprehensive summarized evidence of the determinants and outcomes of eHealth literacy in healthy adults. It encompasses a wide range of factors, both non-modifiable and individual, as well as environmental and cultural aspects. In addition, the review acknowledges that different layers of eHealth literacy determinants can have varying policy implications. This recognition is crucial for policymakers and healthcare professionals seeking to enhance eHealth literacy, as it provides insights into targeted interventions and strategies.

### Study limitations

As with all systematic reviews, we cannot be fully certain that we have identified all relevant literature. This review only covered six databases and studies published in English. We may have missed potentially eligible studies published in other languages or contained within other databases. We only included articles which measured associations of eHealth literacy with the determinant or outcome variables. Articles presenting only differences between those variables were excluded. This constraint might also limit the findings of the review. Furthermore, we limit our population to healthy adults, who may not be the vulnerable population with regards to eHealth literacy. It is important to note that the studies included a relatively diverse group of healthy adults, e.g. students and healthcare professionals. While such inclusion can provide a more comprehensive picture of eHealth literacy in healthy adults, it can also introduce heterogeneity into the study population. Therefore, careful consideration of how the results is interpreted and generalized is needed.

## Conclusions and future directions

This systematic review has contributed to the understanding of the putative determinants and outcomes of eHealth literacy in healthy adults. In summary, the determinants of eHealth literacy include the non-modifiable factors (age, sex); individual factors (literacy, online health information seeking behavior); living and working conditions (education, employment, health care service and ICT infrastructure) and general cultural conditions (particularly language). Whereas, the outcomes of eHealth literacy include health promotion behaviors, COVID-19 preventive behaviors, psychological wellbeing, better social and community networks, better self-rated health and better health service utilisation. Different layers of eHealth literacy determinants can have different policy implications as they aim to improve eHealth literacy.

Future studies should incorporate artificial intelligence (AI) into the eHealth landscape. One possible direction is the expansion of the eHealth Literacy tool to include AI-driven health information. AI can also be used to develop inclusive eHealth solutions that are tailored to different literacy levels. The key to AI’s influence is personalization, which enables the creation of eHealth intervention finely tuned to individual literacy levels. This personalisation encourages more effective health information dissemination while improving accessibility.

## Supporting information

S1 Table Details of search strategy. (PDF)

S2 Table Characteristics of included studies. (PDF)

S3 Table Joanna-Briggs institute critical appraisal for included studies. (PDF)
