# Supplementary material for: Determinants and outcomes of eHealth literacy in healthy adults: A systematic review
Source: PLoS One. 2023 Oct 4;18(10):e0291229. doi: 10.1371/journal.pone.0291229 (PMC10550189; doi:10.1371/journal.pone.0291229)
Supplement: S1 Table — (PDF) [file pone.0291229.s001.pdf]

S1 Table. Details of search strategy

Database	Search strategy
MEDLINE (OVID)	1. "E-health" [tiab] OR "electronic health" [tiab] OR ehealth [tiab] OR "digital health" [tiab] OR "online health" [tiab]<br>2. Literacy [Mesh]<br>3. 1 AND 2<br>4. (determinant$ OR outcome$ OR factor$ OR association OR relationship)<br>5. 3 AND 4
EMBASE	1. 'E-health'/exp OR 'electronic health'/exp OR ehealth/exp OR 'digital health'/exp OR 'online health':ab,ti<br>2. Literacy/exp<br>3. 1 AND 2<br>4. (determinant$ OR outcome$ OR factor$ OR association OR relationship)<br>5. 3 AND 4
PsycInfo	1. "E-health" OR "electronic health" OR ehealth OR "digital health" OR "online health"<br>2. Literacy<br>3. 1 AND 2<br>4. (determinant$ OR outcome$ OR factor$ OR association OR relationship)<br>5. 3 AND 4
CINAHL	1. ("E-health" OR "electronic health" OR ehealth OR "digital health" OR "online health").ab<br>2. Literacy<br>3. 1 AND 2<br>4. (determinant$ OR outcome$ OR factor$ OR association OR relationship)<br>5. 3 AND 4
COCHRANE LIBRARY	[Title Abstract keyword]: "E-health" OR "electronic health" OR ehealth OR "digital health" OR "online health"<br>AND MesH descriptor: Literacy<br>AND [Title Abstract keyword]: (determinant$ OR outcome$ OR factor$ OR association OR relationship)
PROQUEST	("E-health" OR "electronic health" OR ehealth OR "digital health" OR "online health") in AB<br>AND Literacy in AB<br>AND (determinant$ OR outcome$ OR factor$ OR association OR relationship) in AB
