# Supplementary material for: Determinants and outcomes of eHealth literacy in healthy adults: A systematic review
Source: PLoS One. 2023 Oct 4;18(10):e0291229. doi: 10.1371/journal.pone.0291229 (PMC10550189; doi:10.1371/journal.pone.0291229)
Supplement: S2 Table — (PDF) [file pone.0291229.s002.pdf]

S2 Table. Characteristics of included studies

No	First author, Year, Country	Aim of study	Study design	Sample size	Type of participants	Participant characteristics	eHealth literacy instrument	Study findings
1	Abdulai 2021, Ghana	To assess the digital literacy of lay consumers of online COVID-19-related information	Cross-sectional survey	268	Lay people	Males (59.7%); Mean age = 28.42 (SD = 6.98); used the internet daily (94.4%); engaged in social media activities (65%); frequently searched the internet for information related to COVID-19 (17.9%)	eHEALS	Younger age (t = -2.7, $p < 0.01$), being male (t = -2.6, $p < 0.05$), and using internet for education purpose (t = 2.6, $p < 0.05$) were significant predictors of eHealth literacy.
2	Adil, 2021, Pakistan	To explore the cognitive, social, and cultural factors of digital health literacy and e-healthcare services utilization among university students in Pakistan	Qualitative	16	University students	Females (50%)	Not measured	Training in ICTs, Availability and accessibility of ICT resources could promote eHealth literacy.
3	An, 2021, USA	To investigate coronavirus-related eHealth literacy and assess the	Cross-sectional survey	1,074	Lay people	Females (55.6%); mean age = 47.3 (SD = 17.1); white race (69.9%); income	eHEALS	-There was a significant association between eHealth literacy and educational attainment

No	First author, Year, Country	Aim of study	Study design	Sample size	Type of participants	Participant characteristics	eHealth literacy instrument	Study findings
		relationship between eHealth literacy and COVID-19−related knowledge, attitudes, and practices				30,000-74,999 (38.4%); education: postsecondary (some college) (31.6%)		(standardised β = - 0.151 , $p < 0.001$) and income (r=0.087, $p = 0.005$) -MANOVA model showed a significant association between eHealth literacy and COVID-19 knowledge, COVID-19 conspiracy beliefs, COVID-19 preventive behaviors ($F_{3,1013}=20.89$, $P<.001$; Wilks Λ=0.94, partial $\eta^2=0.058$).
4	Amoah, 2021, Hong Kong and Macau, China	To examine the association between DHL (in relation to COVID-19) and psychological well-being of university students during the COVID-19 pandemic	Cross-sectional survey	801	University students	Undergraduate students (68.5%)	Digital Health Literacy Instrument (DHLI)	Digital health literacy showed a positive association with psychological wellbeing (B = 1.115, $p < 0.001$).
5	Britt, 2017, USA	To examine the eHealth literacy of the college student	Cross-sectional survey	420	College students	Mean age = 20.48, SD = 2.14 years;	eHEALS	Future intention to maintain health (r = .451, $p = 0.001$), exercise (r =

No	First author, Year, Country	Aim of study	Study design	Sample size	Type of participants	Participant characteristics	eHealth literacy instrument	Study findings
		health behaviors identified by the American College Health Association				undergraduate students (mean 2.76, SD 1.15); race/ethnicity of white (78.6%)		.380, $p = 0.001$), sleep ($r = .213$, $p = 0.02$), getting vaccinations ($r = .322$, $p = 0.001$), maintenance of sexual health ($r = .332$, $p = 0.001$), balanced diet ($r = .358$, $p = 0.001$), safe sex practices ($r = .332$, $p < 0.01$), stable friendships/social support ($r = .456$, $p = 0.001$), and maintaining a lifestyle free of harmful substances ($r = .333$, $p = 0.001$) were found to have significant correlations with eHealth literacy.
6	Chang, 2021, USA	To examine how information source and consumers' eHealth literacy affect their use of different types of indicators and criteria in evaluating the credibility of	Cross-sectional survey	25	University students	Females (n = 15); mean age = 23.8 years, SD = 5.50; 52% were Caucasian, 28% Hispanic, 12% Asian, 4% African American, and 4% multiple ethnicities	eHEALS	No significant result was noted between the variables.

No	First author, Year, Country	Aim of study	Study design	Sample size	Type of participants	Participant characteristics	eHealth literacy instrument	Study findings
		online health information						
7	Cho Jaehee, 2014, South Korea	To investigate the associations of four cognitive factors: health consciousness, health information orientation, eHealth literacy, and health-app use efficacy with the extent of health-app use	Cross-sectional survey	765	Lay people	Females (49.4%); mean age 37.1 years; held a college degree (67.5%), a high school degree (22.7%), graduate degrees (9%)	eHEALS	eHealth literacy significantly and positively affected health-app use efficacy (beta = 0.39, $p = 0.005$), which ultimately influenced the extent of health-app use (beta = 0.233, $p = 0.023$).
8	Cho Hyeonmi, 2018, South Korea	To examine the associations of eHealth Literacy with health-promoting behaviors among hospital nurses in South Korea	Cross-sectional survey	485	Nurses	Females (98.6%); mean age 30.79 (SD = 6.57); not married 70.7%; held bachelor's degree (48.6); worked at general unit (51.3); worked with night shift (79.4%); monthly income: 2,000-4,000 USD; frequency of internet searching: several times a month	eHEALS	Stress management (γ = 0.12, $p = 0.01$), health promoting behaviors (γ = 0.20, $p < 0.01$), health responsibility (γ = 0.15, $p < 0.01$), and better physical activity among nurses working on fixed-day and day-evening shifts (γ = 0.24, $p = 0.02$) had significant associations with eHealth literacy.

No	First author, Year, Country	Aim of study	Study design	Sample size	Type of participants	Participant characteristics	eHealth literacy instrument	Study findings
						(32.3%); competence in computer usage: moderate (59.6%)		
9	Do, 2020, Vietnam	To identify the associations of HL and eHEALS scores with adherence to infection prevention and control (IPC) procedures, lifestyle changes, and suspected COVID-19 symptoms among HCWs during lockdown	Cross-sectional survey	5,209	Health care professionals	Males (32.9%); 17.4% were aged 41-60 years; 75.2% were ever married; 47.2% could very or fairly easily pay for medication; 86.5% had a middle or high social status; 48.8% were nurses; 44.1% were frontline HCWs	eHEALS	Male gender (B = 0.72, 95% CI 0.43-1.00, $p < 0.001$), having a very or fairly easy ability to pay for medication (B = 0.60, 95% CI 0.34-0.86, $p < 0.001$), having a very or fairly easy ability to pay for doctors (B = 0.56, 95% CI 0.20-0.93, $p = 0.003$) and those with epidemic containment experience (B = 0.64, 95% CI 0.38-0.91, $p < 0.001$) were significantly associated with eHealth literacy.<br><br>Higher eHealth literacy was found to be associated with better adherence to COVID-19 preventive behaviors (B

No	First author, Year, Country	Aim of study	Study design	Sample size	Type of participants	Participant characteristics	eHealth literacy instrument	Study findings
								= 0.22, 95% CI 0.19-0.26, $p < 0.001$), lower likelihood of suspected COVID-19 symptoms (OR = 0.96, 95% CI 0.95-0.98, $p < 0.001$), healthy diet (OR = 1.04, 95% CI 1.02-1.07, $p = 0.002$), and exercise (OR = 1.04, 95% CI 1.03-1.05, $p < 0.001$)
10	Efthymiou, 2021, Cyprus and Greece	To identify the levels of HL and eHL among carers of people with dementia in Greece and Cyprus and to search for the associations with other caring concepts	Cross-sectional survey	174	Carers of people with dementia	Females (76%), over 45 years old (92%), spent on an average of 87 hr of care per week; caring 24/7 (36%); pensioners (44%), socioeconomic position was average (M = 5.86, SD = 1.53)	eHEALS	eHealth literacy score was increased by 0.862 for each point of health literacy score (B = 0.862, 95% CI 0.46 - 1.27).
11	Guo, 2021, Hong Kong, China	To evaluate socioeconomic disparities in eHealth literacy and seeking of	Cross-sectional survey	1,501	Lay people	Females (52.6%); aged ≥ 60 years (27.7%); married or cohabitating (66.1%);	eHEALS	Age and higher education were significantly associated with eHealth literacy score.<br>- Age:

No	First author, Year, Country	Aim of study	Study design	Sample size	Type of participants	Participant characteristics	eHealth literacy instrument	Study findings
		web-based information on COVID-19, and their associations with COVID-19 preventive behaviors				economically active (62.9%) Most participants had attained secondary or tertiary and above education; monthly personal income was HK ≤$10,000 (37.5%); participants self-reported seeking web-based information on COVID-19 (67.8%)		• 40-59 (β = -1.67, p < 0.001)<br>• ≥ 60 (β = -9.24, p < 0.001)<br>- Higher education:<br>• Secondary (β = 7.84, p < 0.001)<br>• Tertiary or above (β = 12.42, p < 0.001)<br><br>eHealth literacy score was associated with higher adherence to COVID-19 preventive behaviors: wearing surgical mask (aOR 1.04, 95% CI 1.01-1.07, p < 0.01)<br>Higher adherence to COVID-19 preventive behaviors: washing hands (aOR 1.06, 95% CI 1.04-1.08, p < 0.001)<br>Higher adherence to COVID-19 preventive behaviors: social distancing (aOR 1.03,

No	First author, Year, Country	Aim of study	Study design	Sample size	Type of participants	Participant characteristics	eHealth literacy instrument	Study findings
								95% CI 1.01-1.05, $p <$ 0.01)<br>Higher adherence to COVID-19 preventive behaviors: bleaching (aOR 1.04, 95% CI 1.02-1.06, $p < 0.01$)
12	Hayat, 2017, Israel	To examine the interpersonal sources and their interplay with respondents' eHealth literacy and perceived health outcomes	Cross-sectional survey	819	Lay people	*Israeli Jews:*<br>Mean age = 51.1 (SD 17.2);<br>Males (48.0%);<br>had internet access (80.4%);<br>education high school or less (44.2%)<br>*Palestinian Citizen of Israel:*<br>Mean age = 42.5 (SD 13.6);<br>Males (49.3%);<br>education high school or less (48.6%)	eHEALS	There was a significant association between eHealth literacy and perceived health outcomes (β=–0.54, p = 0.003)
13	Holt, 2020, Denmark	To examine the level of three literacies (health literacy, digital	Cross-sectional survey	366	Nursing students	Females (92.0%);<br>Mean age = 24.6 (IQR 21–25) years among entry-level	eHLQ	Students' educational levels before entering the nursing program were correlated only with

No	First author, Year, Country	Aim of study	Study design	Sample size	Type of participants	Participant characteristics	eHealth literacy instrument	Study findings
		literacy, and eHealth literacy) in entry- and graduate-level nursing students and examine sociodemographic characteristics and self-rated health associations				students and 26.8 (IQR 24–28) years among graduate-level students; reported that they suffered from a chronic condition (21%); took prescribed medication daily (57.1%)		eHLA3, familiarity with health and health care (tau-b = .187, p = .013). Parental educational levels were positively, moderate correlated with eHLQ2, understanding of health concepts and language (tau-b = .211, p = .003) and weakly correlated to eHLQ3, ability to actively engage with digital services (tau-b = .139, p = .043).
14	Huang, 2020, Taiwan	To investigate the associations between individual factors, electronic health (eHealth) literacy, dietary behaviors, and exercise habits in college students	Cross-sectional survey	674	College students	Mean age = 20.44 years (SD = 2.03); 56.5% took dietary supplements in their daily lives; 43.3% in good or very good subjective health status; 48.2% considered health to be important or very important	eHLS (eHealth Literacy Scale)	eHealth literacy could predict dietary behaviors and exercise habits with an overall explanatory power of 13% ($F = 8.91$, $p < 0.001$) and 16% ($F = 10.86$, $p < 0.001$), respectively.

No	First author, Year, Country	Aim of study	Study design	Sample size	Type of participants	Participant characteristics	eHealth literacy instrument	Study findings
15	Kim Sisook, 2020, South Korea	To identify the eHealth literacy levels and the factors affecting eHealth literacy among nursing students	Cross-sectional survey	205	Nursing students	Female (85.9%); Mean age = 21.69 (SD = 2.77); had grown up in a major city (94.6%)	eHEALS	eHealth literacy was significantly associated with academic level (β = 0.15, $p = 0.012$), digital literacy towards ICT for learning (β = 0.40, $p < 0.001$), and self-efficacy for online education (β = 0.22, $p = 0.002$).
16	Kim Sun-hee, 2017, South Korea	To examine the the relationship between eHealth literacy and health behaviors	Cross-sectional survey	230	Lay people	Females (57.8%); aged 18-29 years (86.1%); Unemployed (80.9%); Single (89.6%); Good perceived health status (49.6); did not have a chronic disease (90.4%).	eHEALS	eHealth literacy was the strongest predictor of health promoting behaviors (β =0.28, $p < 0.001$), after adjusting for sociodemographic and health-related characteristics.
17	Kim Sunghee, 2021, South Korea	To explore possible multistep and indirect pathways of association between e-health literacy and health-promoting	Cross-sectional survey	558	Nursing students	Female (88.4%); Mean age = 20.3 years (SD = 2.2); unaffiliated with religious groups (60.8%); 31.0% of participants used the	eHEALS	eHealth literacy showed significant positive correlations with online health information seeking behavior (r = 0.25, $p < 0.001$), health promoting behaviors (r = 0.37, $p < 0.001$), and

No	First author, Year, Country	Aim of study	Study design	Sample size	Type of participants	Participant characteristics	eHealth literacy instrument	Study findings
		behaviors through social media use for health information, online health information-seeking behaviors, and self-care agency among nursing students				internet for less than two hours per day on average; 74.7% of participants reported that they spent more than four days per week searching for health-related information online; having a good perceived health status (55.4%)		self-care agency/self-care competency (r = 0.45, $p < 0.001$).
18	Knapp, 2011, USA	To describe eHealth literacy of Internet users, and to determine which child and household factors were associated with greater eHealth literacy	Cross-sectional survey	2,371	Parents of children with special health care needs	Females (91%); white non-Hispanic (39%); had a high school diploma (30%) English speaking (77%); high school graduates (30%); married (53%); living in a two-parent household (51%); used the Internet (82%) 49% of those parents used it daily	eHEALS	Non-English language, lower educational attainment, and older age were significantly associated with lower levels agreement with the eight eHEALS statement.

No	First author, Year, Country	Aim of study	Study design	Sample size	Type of participants	Participant characteristics	eHealth literacy instrument	Study findings
19	Kritsotakis, 2020, Greece	To examine eHealth literacy levels in nurses and to explore its associations with the nursing practice environment	Cross-sectional survey	200	Nurses	Female (91%); age between 45-54 years 35%); middle-level financial status (66%); 60.5% were nurses and 39.5% assistant nurses; 49.5% had a 4-year university-level degree; 79% had leadership status; 49% worked in the current department for less than five years	eHEALS	Higher scores on "collegial nurse-physician relationships" (OR = 2.04, 95% CI 1.21–3.44, $p \leq 0.05$) and "nurse participation in hospital affairs" (OR = 2.03, 95% CI 1.08–3.79, $p \leq 0.05$) were associated with higher eHEALS scores.
20	Li Shaojie, 2021, China	To examine the associations between COVID-19 behaviors and participants' health literacy and eHealth literacy	Cross-sectional survey	1,873	College students	Males (51.7%); mean age = 19.6 years (SD 1.8); rural residence (53.5%); medium family economic level (59.5%); (80.4%) had good self-reported health status	eHEALS	eHealth literacy was significantly associated with COVID-19 specific precautionary behaviors ($\beta = 0.368$, $p < 0.001$) and conventional health behaviors ($\beta = 0.219$, $p < 0.001$)

No	First author, Year, Country	Aim of study	Study design	Sample size	Type of participants	Participant characteristics	eHealth literacy instrument	Study findings
21	Li Xiaojing, 2020, China	To examine the predictive role of social media use on public preventive behaviors in China during the COVID-19 pandemic	Cross-sectional survey	802	Lay people	Males (51.9%); ages ranged from 20 to 60 years; above bachelor's degree (77.7%); high monthly income of >¥5000 (65.3%); married (61.8%); in good health (60.6%)	eHEALS	eHealth literacy significantly predicted higher adherence to COVID-19 preventive measures (β=.27, $p <$ 0.001)
22	Luo, 2018, Taiwan	To examine the associations among sex, eHealth literacy, and health services utilisation	Cross-sectional survey	489	College students	Mean (SD) age = 21.51 (4.11) year	eHLS	Interactive and critical eHealth literacy were positively correlated to health service utilization.
23	Lwin, 2020, China	To examine the determinants of both self-efficacy in utilizing eHealth and frequency of eHealth information evaluation	Cross-sectional survey	923	Lay people	Males (48.1%); Aged between 21 and 35 (67.2%); held at least a bachelor's degree (77.2%); had a household income of more than ￥12,000 (59.5%); reported that they used the Internet a few times a day (71.3%);	eHEALS	Age (r = 0.12, $p < 0.001$), higher education (r = 0.15, $p < 0.001$), health information orientation (r = 0.65, $p < 0.001$), and internet literacy (r = 0.49, $p < 0.001$) was positively associated with eHealth literacy<br>Males had lower eHealth literacy than females (β = −0.14, p = 0.001). Those with higher monthly

No	First author, Year, Country	Aim of study	Study design	Sample size	Type of participants	Participant characteristics	eHealth literacy instrument	Study findings
						possessed good to excellent Internet skills (69.6%).		household income had higher eHealth literacy than those with less household income (β = 0.21, *p* < 0.001).
24	Magsamen-Conrad, 2019, USA	To assess generational differences in New Communication Technology (NCT) use and eHealth literacy	Cross-sectional survey	525	Lay people	Mean age = 50.01 (SD= 16.48); Caucasian (85%); U.S. citizenship (94%); Years in school, mean = 15.83 (SD= 2.72); had searched for health information online at some point (80%)	eHEALS	Level of education (β = 0.12, *p* < 0.001), online health information searching experience (β = 0.13, *p* < 0.001), New Communication Technology (NCT) Effort expectancy (β = 0.40, *p* < 0.001), and NCT Performance expectancy (β = 0.24, *p* < 0.001) significantly predicted eHealth literacy.
25	Mitsutake, 2012, Japan	To assess the associations between eHealth literacy, knowledge of colorectal cancer (CRC), and CRC screening practices	Cross-sectional survey	2,970	Lay people	Males (49.9%); mean age (SD) = 39.7 (10.9) years; married (60.9%); had graduated from college or graduate school (51.6%); used	eHEALS	eHealth literacy was positively associated with CRC screening knowledge (β = .116, p < 0.001). After controlling for age, marital status, education level, and household income level,

No	First author, Year, Country	Aim of study	Study design	Sample size	Type of participants	Participant characteristics	eHealth literacy instrument	Study findings
						the internet every day (70%)		a I point increase in the eHEALS score signified that the individuals were 1.03 times (95% CI 1.01–1.05) more likely to undergo CRC screening practice.
26	Mitsutake, 2016, Japan	To examine the association between eHealth literacy and general health behavior (cigarette smoking, physical exercise, alcohol consumption, sleeping hours, eating breakfast, eating between meals, and balanced nutrition)	Cross-sectional survey	10,178	Lay people	Males (49.74%); mean age (SD) = 39.7 (10.9) years; had graduated from college or graduate school (50.69%); used the Internet every day (72.06%)	eHEALS	Individuals with high eHealth literacy were more likely to perform exercise (AOR= 1.377, 95% CI 1.131-1.678) and eating a balanced diet (AOR 1.572, 95% CI 1.274-1.940) than individuals with low eHealth literacy.
27	Neter, 2021, Israel	To identify group differences (ethnicity, immigration) in eHealth literacy	Cross-sectional survey	819	Lay people	Females (51.85%); Mean age = 49.72 ± 17.00; elementary to full secondary education (45%); internet use (73.3%)	eHEALS	eHealth literacy was significantly correlated to self-rated health ($r=0.13$, $p = 0.01$) and to outcomes gained from the internet search ($r = 0.40$, $p = 0.000$).

No	First author, Year, Country	Aim of study	Study design	Sample size	Type of participants	Participant characteristics	eHealth literacy instrument	Study findings
28	Ozkan, 2022, Turkey	to identify the relationship between e-health literacy, psychological well-being, and perceived coronavirus disease threat	Cross-sectional survey	5,153	Lay people	Females (68.9%); aged 18-25 (61.7%); had undergraduate degree (63.5%); single (68.0%); lived in metropolitan cities (52.3%); did not work (64%); monthly income level 0-1000 TL (39.2%); did not have any chronic illness (87.9%)	eHEALS	eHealth literacy was significantly associated with psychological well-being (β = 0.432; $p < 0.01$).
29	Park, 2014, USA	To examine relationships between level of eHealth literacy and the experience of seeking cancer information, to identify the association between eHealth literacy level and educational needs about searching cancer information from the Internet,	Cross-sectional survey	108	Lay people	Females (52%) Aged 50 to 59 years (31.5%); Caucasian (51.9%), graduate from a 2-4 year college or graduate school (77.8%); married (50%); had a household income less than US$25000 (31.5%); more than 60% of the respondents considered their health status to be	eHEALS	eHealth literacy was associated with more confidence in finding cancer information ($p < 0.05$) and needing more education about cancer screening tests ($p < 0.05$).

No	First author, Year, Country	Aim of study	Study design	Sample size	Type of participants	Participant characteristics	eHealth literacy instrument	Study findings
		and to assess the relationship between eHealth literacy level and prior experience with cancer screening				excellent or very good; 65% had a history of cancer in their family		
30	Paige, Krieger, & Stellefson, 2017a, USA	To examine the relationship between eHealth literacy and perceived trust in online health communication channels and sources among diverse sociodemographic groups	Cross-sectional survey	811	Lay people	Mean age =46.3 years, SD = 17.2 years; females (72.6%); Caucasian (50.4%); Black/AA (49.6%); 44% earned $50,000 or more annually; 75% reported at least some college education. Using Facebook (55.6%), to locate or share health information	eHEALS	Sex (Male) (b= -0.35, $p < 0.05$) and age (b = 0.01, $p < 0.05$) were associated with eHealth literacy.<br><br>Greater eHealth literacy significantly predicted higher perceived trust in online information (b = 0.25, $p < 0.05$).
31	Paige, Stellefson, et al., 2017b, USA	To assess how intensity of Instagram use moderates the relationship between eHealth	Cross-sectional survey	327	College students	Females (50.8%); mean age = 24 years (SD = 7 years); Caucasian (64.8%)	eHEALS	Intensity of Instagram use ($F(8, 194) = 4.95$, $p < 0.001$) and online bridging social capital (b = 0.20; $p < 0.05$) were

No	First author, Year, Country	Aim of study	Study design	Sample size	Type of participants	Participant characteristics	eHealth literacy instrument	Study findings
		Literacy and online social capital						associated with eHealth literacy.
32	Qin, 2022, China	To examine the associations of COVID-19 risk perception, eHealth literacy, and protective behaviors for Chinese college students following vaccination	Cross-sectional survey	5,641	College students	Males (59.01%); mean age = 21.39 years (SD = 2.75); rated their health as very good (44.85%) or pretty good (46.98%)	eHEALS	eHealth literacy was associated with the level of COVID-19 preventive behaviors (B = 0.225, $p < 0.001$).
33	Quinn, 2017, UK	To assess the associations between health literacy, eHealth literacy and actual online health information seeking behavior	Cross-sectional simulation	54	Lay people	Females (38.9%); mean age = 26.76 years (SD = 9.64); 62.9% held an undergraduate or postgraduate degree; from computer science background 70.3%	eHEALS	No significant results
34	Sarkar, 2016, USA	To examine the relationships between the psychosocial health of caregivers of children with special healthcare	Cross-sectional survey	313	Caregivers of children with special healthcare needs	Females (90%); biological mother of the child (84%); white (67.1%); had some college education (62.9%);	eHEALS	Older age (r = -0.185, $p = 0.001$) was associated with lower eHEALS scores.<br>Higher education (β = 0.12, $p < .001$) was

No	First author, Year, Country	Aim of study	Study design	Sample size	Type of participants	Participant characteristics	eHealth literacy instrument	Study findings
		needs and their e-health use				between age 41-60 years (50%)		associated with higher eHEALS scores.
35	Shiferaw, 2019, Ethiopia	To assess the extent of Internet use and eHealth literacy among a cross section of health-care professionals	Cross-sectional survey	291	Health care professionals	Females (53.7%); mean age = 30.09 (SD = 5.025) years; held a bachelor's degree (76.7%); Nurses (30.7%); Less than 5 years of work experience (58.2%)	eHEALS	Health care professionals aged 20-29 years, occupation: physician (r = 1.889, $p = 0.017$), and those with years of experience < 5 years (r = 1.392, $p = 0.000$) were more likely to have higher eHealth literacy.
36	Shiferaw, 2020, Ethiopia	To assess digital competency of healthcare providers	Cross-sectional survey	167	Health care professionals	Males (52.7%); mean age = 28.2 (SD = 5.5); held a diploma degree 55.7%; 90.7% nurses; mean work experience 4.6 years (SD = 3.9); monthly income 3501-5500 Ethiopian birr 64.1%	European Comission's digital competency framework	Sex (AOR = 3.914, 95% CI 1.745-8.776, $p = 0.001$), higher education (AOR = 0.366, 95% CI 0.149-0.900, $p < 0.029$), higher income (AOR = 0.105, 95% CI 0.105-0.923, $p = 0.042$), and occupation: Government employee occupation (AOR: 0.048, 95% CI 0.005-0.423, $p = 0.006$) was associated with higher eHealth literacy.

No	First author, Year, Country	Aim of study	Study design	Sample size	Type of participants	Participant characteristics	eHealth literacy instrument	Study findings
37	Suri, 2016, Singapore	To identify the relationship between five domain-specific skills of health literacy: Find Health Information (FHI), Appraise Health Information (AHI), Understand Health Information to act (UHI), Actively Manage One's Health (AMH), and E-health literacy (e-Heals), and health information seeking behaviors and three categories of health outcomes	Cross-sectional survey	1062	College students	Females (53.7%); age 18-22 years (73.6%); parents' highest level of education: undergraduate degree (27.0%); race and ethnicity: Chinese (83.1%); good health status (40.6%); BMI healthy (63.4%)	eHEALS	Online health information seeking behavior ($\beta = 0.12$, $p < .001$) was associated with eHealth literacy.
38	Tsukahara, 2020, Japan	To examine the eHealth literacy level, the participant characteristics associated with	Cross-sectional survey	3183	University students	Males (72.4%); year 4 undergraduate students (19.3%); living alone (50.2%)	eHEALS	Individuals with higher eHealth literacy were more likely to perform regular exercise (OR = 1.42, 95% CI 1.23-1.63, $p < 0.001$),

No	First author, Year, Country	Aim of study	Study design	Sample size	Type of participants	Participant characteristics	eHealth literacy instrument	Study findings
		eHealth literacy, and the association of eHealth literacy with lifestyle behaviors of students						regular breakfast (OR = 1.18, 95% CI 1.02-1.37, $p < 0.001$), and had lower risk of being overweight (OR = 1.58, 95% CI 1.23-2.05, $p < 0.001$).
39	Xesfingi, 2016, Greece	To assess the eHealth literacy level of Greek citizens, and the associated factors	Cross-sectional survey	1064	Lay people	Females (55.1%); Single (51.6%); university education (48.5%)	eHEALS	Age (OR = 0.771, $p < 0.001$), higher education (OR = 1530, $p < 0.001$), computer literacy (OR = 2.568, $p < 0.001$), information literacy (OR = 3.072, $p < 0.001$), and exercise (OR = 1.540, $p < 0.001$) were associated with eHealth literacy.
40	Yang, Shu-Ching, 2017, Taiwan	To identify the associations among various individual factors, eHealth literacy, and health-promoting lifestyles	Cross-sectional survey	556	College students	Females (80.9%); major in nonmedical field (80.9%); average degree of health concern (43.5%); Frequency of seeking information on health-related issues: sometimes (48.6%)	Chiang's EHLS	Critical eHealth literacy positively predicted all 6 health-promoting lifestyle dimensions: (1) self-actualisation, (2) health responsibility, (3) interpersonal support, (4) exercise, (5) nutrition, (6) stress management (t_{547}=2.66-7.28, P<.01),

No	First author, Year, Country	Aim of study	Study design	Sample size	Type of participants	Participant characteristics	eHealth literacy instrument	Study findings
								functional literacy positively predicted 2 dimensions (t547=2.32-2.98, P<.05), and interactive literacy predicted only the self-actualization dimension (t547=2.81, P<.01)
41	Yang, Shu-Ching, 2019, Taiwan	To examine whether individual differences and higher eHealth literacy are associated with more positive dietary behaviors and less unhealthy dietary intake	Cross-sectional survey	813	College students	Females (47.1%); mean age 20.08 years (SD = 1.43); monthly expenses NT $ 5001-NT $10,000 (503%);	Chiang's EHLS	Functional eHealth literacy was positively associated with balanced diet (β=.25; $p < 0.001$), negatively associated with unhealthy food intake (β=−.11; $p = 0.01$), and consumer health (β=0.15; $p = 0.02$).<br><br>Critical eHealth literacy was positively associated with consumer health (β=0.30; $p < 0.001$) and regular eating habit (β=0.20; $p = 0.002$).
42	Yang, Bing Xiang, 2021, China	To assess the eHealth literacy and the psychological	Cross-sectional survey	15000	Lay people	Females (57.1%); Aged between 31-40 (42.5%); bachelor's	eHealth Literacy Questionnaire (EHLQ)	eHealth literacy was negatively correlated with depression (r = −0.331, $p < .001$),

No	First author, Year, Country	Aim of study	Study design	Sample size	Type of participants	Participant characteristics	eHealth literacy instrument	Study findings
		status of Chinese residents during the COVID-19 pandemic and explore their interrelationship				degree/college (68.3%)		insomnia (r = −0.366, *p* < .001), and post-traumatic stress disorder (r = −0.320, *p* < .001).
43	Yuan, 2020, China	To examine the association between eHealth literacy and COVID-19 prevention behavior among nursing students in China	Cross-sectional survey	613	Nursing student	Males (31.8%); mean age = 20.88 years (SD = 1.55); lived in the city (35.8%)	eHEALS	eHealth literacy was significantly correlated with higher adherence to COVID-19 preventive measures (rs = 0.416, *p* < 0.01)
44	Zakar, 2021, Pakistan	To assess information seeking behaviors, as well as the ability to find relevant information and deal with digital health literacy	Cross-sectional survey	1747	University students	Female (52.7%); mean age = 22.5 years (SD = 4.5); education: bachelors' degree (52.4%); semester currently studying: 1-2 (43.4%); subjective social status: medium (49.9%); primary source of financing:	DHLI	Sex (female) (AOR = -0.02, *p* = 0.005), sense of coherence (Beta = 0.13 (SE= 0.02), *p* < 0.001), satisfaction with information (Beta = −1.05 (SE = 0.23), *p* < 0.001), and importance of information (Beta = 2.02 (SE = 0.33), *p* < 0.001) were significantly associated with digital health literacy.

No	First author, Year, Country	Aim of study	Study design	Sample size	Type of participants	Participant characteristics	eHealth literacy instrument	Study findings
						support by parents (76.8%)		
