# Supplementary material for: Determinants and outcomes of eHealth literacy in healthy adults: A systematic review
Source: PLoS One. 2023 Oct 4;18(10):e0291229. doi: 10.1371/journal.pone.0291229 (PMC10550189; doi:10.1371/journal.pone.0291229)
Supplement: S3 Table — (PDF) [file pone.0291229.s003.pdf]

S3 Table. Joanna-Briggs Institute critical appraisal for cross-sectional studies

No	First author, year	1	2	3	4	5	6	7	8
1	Abdulai, 2021	Yes	Yes	Yes	Yes	No	No	Yes	Yes
2	Amoah, 2021	Yes	Yes	Yes	Yes	Yes	Yes	Yes	Yes
3	An, 2021	No	No	Yes	Yes	Yes	Yes	Yes	Yes
4	Britt, 2017	No	Yes	Yes	Yes	No	No	No	No
5	Chang, 2021	Yes	Yes	Yes	Yes	No	No	Yes	Yes
6	Cho Jaehee, 2014	Yes	Yes	Yes	Yes	No	No	Yes	Yes
7	Cho Hyeonmi, 2018	Yes	Yes	Yes	Yes	Yes	Yes	Yes	Yes
8	Do, 2020	No	Yes	Yes	Yes	No	No	Yes	Yes
9	Efthymiou, 2020	Yes	Yes	Yes	Yes	Yes	Yes	Yes	Yes
10	Guo, 2021	Yes	Yes	Yes	Yes	No	No	No	Yes
11	Hayat, 2017	Yes	Yes	Yes	Yes	Yes	Yes	Yes	Yes
12	Holt, 2020	Yes	Yes	Yes	Yes	No	No	Yes	Yes
13	Huang, 2020	No	Yes	Yes	Yes	No	No	Yes	Yes
14	Kim Sisook, 2020	Yes	Yes	Yes	Yes	No	No	Yes	Yes
15	Kim Sun-hee, 2017	Yes	Yes	Yes	Yes	No	No	Yes	Yes
16	Kim Sunghee, 2021	No	No	Yes	Yes	No	No	No	Yes
17	Knapp, 2011	Yes	Yes	Yes	Yes	No	No	Yes	Yes
18	Kritsotakis, 2020	No	No	Yes	Yes	No	No	Yes	Yes
19	Li Shaojie, 2021	Yes	Yes	Yes	Yes	Yes	Yes	Yes	Yes
20	Li Xiaojing, 2020	Yes	Yes	Yes	Yes	No	No	Yes	Yes
21	Luo, 2018	Yes	Yes	Yes	Yes	No	No	Yes	Yes
22	Lwin, 2020	Yes	Yes	Yes	Yes	No	No	Yes	Yes
23	Magsamen-Conrad, 2019	Yes	Yes	Yes	Yes	No	No	Yes	Yes
24	Mitsutake, 2012	Yes	Yes	Yes	Yes	Yes	Yes	Yes	Yes
25	Mitsutake, 2016	Yes	Yes	Yes	Yes	Yes	Yes	Yes	Yes
26	Neter, 2021	Yes	Yes	Yes	Yes	No	No	Yes	Yes
27	Ozkan, 2022	Yes	Yes	Yes	Yes	No	No	Yes	Yes
28	Paige, 2017a	Yes	No	Yes	Yes	Yes	Yes	Yes	Yes

No	First author, year	1	2	3	4	5	6	7	8
29	Paige, 2017b	Yes	Yes	Yes	Yes	Yes	Yes	Yes	Yes
30	Park, 2014	Yes	Yes	Yes	Yes	No	No	Yes	Yes
31	Qin, 2022	Yes	Yes	Yes	Yes	No	No	Yes	Yes
32	Quinn, 2017	No	Yes	Yes	Yes	No	No	Yes	Yes
33	Sarkar, 2016	Yes	Yes	Yes	Yes	Yes	Yes	Yes	Yes
34	Shiferaw, 2019	Yes	Yes	Yes	Yes	No	No	Yes	Yes
35	Shiferaw, 2020	Yes	Yes	Yes	Yes	No	No	Yes	Yes
36	Suri, 2016	Yes	Yes	Yes	Yes	No	No	Yes	Yes
37	Tsukahara, 2020	Yes	Yes	Yes	Yes	No	No	Yes	Yes
38	Xesfingi, 2016	Yes	Yes	Yes	Yes	Yes	Yes	No	Yes
39	Yang, 2017	No	Yes	Yes	Yes	No	No	Yes	Yes
40	Yang, 2019	No	Yes	Yes	Yes	No	No	Yes	Yes
41	Yang, 2021	Yes	Yes	Yes	Yes	No	No	Yes	Yes
42	Yuan, 2020	Yes	Yes	Yes	Yes	No	No	Yes	Yes
43	Zakar, 2021	Yes	Yes	Yes	Yes	No	No	Yes	Yes

The Joanna-Briggs Institute critical appraisal for cross-sectional studies:

1. Were the criteria for inclusion in the sample clearly defined?
2. Were the study subjects and the setting described in detail?
3. Was the exposure measured in a valid and reliable way?
4. Were objective, standard criteria used for measurement of the condition?
5. Were confounding factors identified?
6. Were strategies to deal with confounding factors stated?

7. Were the outcomes measured in a valid and reliable way?
8. Was appropriate statistical analysis used?

S4 Table. Joanna-Briggs Institute critical appraisal for qualitative studies

No	First Author, year	1	2	3	4	5	6	7	8	9	10
1	Adil, 2021	Yes	Yes	Yes	Yes	Yes	No	No	Yes	No	Yes

The Joanna-Briggs Institute critical appraisal for qualitative studies:

1. Is there congruity between the stated philosophical perspective and the research methodology?
2. Is there congruity between the research methodology and the research question or objectives?
3. Is there congruity between the research methodology and the methods used to collect data?
4. Is there congruity between the research methodology and the representation and analysis of data?
5. Is there congruity between the research methodology and the interpretation of results?
6. Is there a statement locating the researcher culturally or theoretically?
7. Is the influence of the researcher on the research, and vice- versa, addressed?
8. Are participants, and their voices, adequately represented?
9. Is the research ethical according to current criteria or, for recent studies, and is there evidence of ethical approval by an appropriate body?
10. Do the conclusions drawn in the research report flow from the analysis, or interpretation, of the data?
