# Supplementary material for: Determinants and outcomes of eHealth literacy in healthy adults: A systematic review
Source: PLoS One. 2023 Oct 4;18(10):e0291229. doi: 10.1371/journal.pone.0291229 (PMC10550189; doi:10.1371/journal.pone.0291229)
Supplement: Attachment — Submitted filename: Response to reviewer_26042023.docx [file pone.0291229.s004.docx]

Dear Editors and Reviewers:

On behalf of all authors, I would like to thank the editor and all reviewers for the valuable feedback that we have used to improve the quality of our manuscript. The reviewer comments are listed below in italicised font, while our responses are given in normal font. Changes or additions we made in the manuscripts are marked with red colour.

**List of response**

**Reviewer #1:**

*Thank you for the opportunity to read this paper. It address an important topic and contributes to the body of knowledge in this field. I only have few minor comments for authors' consideration.*

*Introduction
1. Please elaborate why the authors want to understand eHealth literacy in healthy adults?*

Reply:

Thank you for your valuable suggestion. We have taken your feedback into consideration and have elaborated on our rationale for studying eHealth literacy in healthy adults. We have included our response in the introduction section of our manuscript, specifically in the last paragraph.

Understanding the determinants and outcomes of eHealth literacy can provide valuable insights into the factors that influence individuals’ ability to use technology to access, understand, and use health information, and the impact that eHealth literacy has on health outcomes. This information can then be used to develop interventions and policies to promote eHealth literacy and improve health outcomes in healthy adults. Focusing on the adult population is important since this population makes up the largest age group in the world [28]. Moreover, by focusing the study population on healthy adults, the researchers can minimise the potential confounding effects of health conditions or other factors that may influence eHealth literacy. (page 2-3)

*2. Why it is important to understand determinants and outcomes?*

Reply:

Thank you for your comment. We believe that understanding the determinants and outcomes of eHealth literacy is crucial because it can help identify the factors that contribute to individuals' ability to access and use health information through technology, and how this ability impacts their health outcomes. By examining these factors, we can develop interventions and policies that promote eHealth literacy and improve health outcomes in healthy adults.

We have added this explanation to the last paragraph of our introduction section.

Understanding the determinants and outcomes of eHealth literacy can provide valuable insights into the factors that influence individuals’ ability to use technology to access, understand, and use health information, and the impact that eHealth literacy has on health outcomes. This information can then be used to develop interventions and policies to promote eHealth literacy and improve health outcomes in healthy adults. Focusing on the adult population is important since this population makes up the largest age group in the world [28]. Moreover, by focusing the study population on healthy adults, the researchers can minimise the potential confounding effects of health conditions or other factors that may influence eHealth literacy. (page 2-3)

*Results
3. It is worth noting that studies included a wide range of participants (e.g. carers or healthcare professionals), please comment on how it may affect the results of this SR.*

Reply:

Thank you for raising this issue. We agree that it is important to note about the wide range of participants which may influence the results of our SR. We have acknowledged it in the following comment at the end of the discussion section.

It is important to note that the studies included a diverse kind of healthy adults, e.g. students and healthcare professionals. While such inclusion can provide a more comprehensive picture of eHealth literacy in healthy adults, it can also introduce heterogeneity into the study population. Therefore, careful consideration of how the results are interpreted and generalized is needed. (page 44)

*Discussion
4. I think readers may be interested to whether the results of this SR in healthy adults differ from those in other populations (e.g. older adults). Please consider adding few sentences to elaborate.*

Reply:

Thank you for your suggestion. We have written an additional paragraph in the discussion section to elaborate the similarities and differences between our SR and the most relevant previous SR.

A systematic review on the influencing factors of eHealth literacy among Chinese older adults [8] showed several similar findings with our study results, i.e. age, gender, education, socioeconomic status, and language influenced the levels of eHealth literacy [8]. These similarities suggest the factors which may be the universal determinants of eHealth literacy across different culture and population. However, our present study found that eHealth literacy was also influenced by literacy factors and ICT infrastructure. Another difference is that physical and psychological conditions, marital status, and being the family carer were found to be associated with the Chinese older adults’ levels of eHealth literacy [8]. These differences may indicate the factors that may be unique to older adult population, especially in China. With regards to outcomes, similar to our study findings, a recent systematic review concluded that eHealth literacy is associated with health-promoting behaviours including regular eating and exercise, and compliance with disease prevention behaviours [27]. These similar and different findings show the importance of conducting systematic review to find the consistent and inconsistent patterns across different studies. (page 43)

**Reviewer #2:**

*This is an interesting review which examines the determinants and outcomes of eHealth literacy.

Introduction/Background
1. Rationale: given that there are already three reviews that examine interventions for health literacy, it would be important to be more specific about how this current review adds to the already extensive reviews in the area, in particular as you state that the results are ‘putative’ and other reviews have focused specifically on interventions and the outcomes of interventions.*

Reply:

Thank you for your meaningful feedback on our manuscript. We agree that it is important to clarify how our review adds to the existing literature on eHealth literacy, given the extensive previous reviews particularly on eHealth literacy interventions for older adults.

Our review takes a broader approach by examining the determinants and outcomes of eHealth literacy in healthy adults, while previous reviews on eHealth literacy interventions focused on older adults. Focusing on the adult population is important since this population makes up the largest age group in the world. Moreover, by focusing the study population on healthy adults, the researchers can minimise the potential confounding effects of health conditions or other factors that may influence eHealth literacy. In addition, our review offers a model of eHealth literacy determinants and outcomes which can be helpful to outline strategic approaches to promote greater equity in eHealth literacy through different policy levels. Finally, our present review found the factors unique to healthy adults which are not found among older adult population as well as the similar factors across these populations (as illustrated in the second last paragraph in the discussion section).

We also added an explanation about this rationale to the last paragraph of our introduction:

“However, to the best of our knowledge, there is still no systematic review to summarise the evidence of eHealth literacy’s determinants and outcomes, unrelated to a specific intervention. To address this perceived gap, this systematic review aims to summarise and appraise the existing research which has examined the determinants and outcomes of eHealth literacy in healthy adults. Understanding the determinants and outcomes of eHealth literacy can provide valuable insights into the factors that influence individuals’ ability to use technology to access, understand, and use health information, and the impact that eHealth literacy has on health outcomes. This information can then be used to develop interventions and policies to promote eHealth literacy and improve health outcomes in healthy adults. Focusing on the adult population is important since this population makes up the largest age group in the world [28]. Moreover, by focusing the study population on healthy adults, the researchers can minimise the potential confounding effects of health conditions or other factors that may influence eHealth literacy.” (page 2-3)

*2. A recently published systematic review:* [*https://pubmed.ncbi.nlm.nih.gov/36716080/*](https://pubmed.ncbi.nlm.nih.gov/36716080/)

Reply:

Thank you for suggesting a recently published SR. We have incorporated it in our introduction (page 2) and discussion sections (page 43).

*3. Why focus on healthy adults only and how are healthy adults defined?*

Reply:

Thank you for your questions. We added these sentences to the end of our introduction section to answer your question:

“Focusing on the adult population is important since this population makes up the largest age group in the world [2]. Moreover, by focusing the study population on healthy adults, the researchers can minimise the potential confounding effects of health conditions or other factors that may influence eHealth literacy.” (page 3)

Also, we have inserted the definition of healthy adults in the eligibility criteria, in the Methods section.

Healthy adults are defined as individuals aged over 18-64 years (American Medical Association, 2022) without significant chronic health conditions or disabilities. (page 3)

*4. Rationale for how ‘outcomes’ can be described as benefits and disadvantages.*

Reply:

Thank you for raising this issue.

We replaced the use of terms ‘benefits and disadvantages’ with positive and negative outcomes (page 3). Describing outcomes as both positive and negative allows for a more balanced assessment of the eHealth literacy topic.

*Methods:*
*5. What software was used to screen the articles?*

Reply:

We used EndNote 20 software to screen the articles (we have added this information in our Methods section, page 3).

*6. Where were the data extracted to?*

Reply:

The data were extracted to an extraction sheet in Microsoft Word (we have added this information in our Methods section page 4).

*7. Data Synthesis: the Dahlgren and Whitehead model is explained, but not how the data were synthesised and applied to this model. How was this undertaken from a methods perspective?*

Reply:

Thank you for raising this issue.

From a methods perspective, we synthesised the data from the included studies to this model by extracting the information on the determinants and outcomes of eHealth literacy and mapping the findings onto the model. Finally we identified areas where interventions or policies can be targeted related to each determinants. (We have incorporated these information at the end of the Data Synthesis part, Methods section)

Data from the included studies were synthesised and applied to this model by extracting the information on the determinants and outcomes of eHealth literacy and mapping the findings onto the layers of Dahlgren and Whitehead’s model. Finally, areas where interventions or policies can be targeted related to each determinant were identified. (page 4)

*8. I don’t think Table 2 or Table 3 are necessary. These could be put in a supplementary file. Also, no need to list the JBI critical appraisal questions.*

Reply:

Thank you for your feedback. We have put table 2 and 3 in the Supplementary material.

*Results/Discussion
9. I would question the appropriateness or need to use of the term ‘putative’ throughout the article. If there is a description of what is meant by putative and why these studies are putative determinants and outcomes only in the background (or early results) and perhaps again in the conclusion, there is no need to repeat this throughout the article.*

Reply:

Thank you for your suggestion. We have removed the term ‘putative’, except in the early part of the Results section (As the causal relationship could not be drawn from the cross-sectional study design, the determinants and outcomes were putative only. Page 5) and in the Conclusions.

*10. Conceptual model of ehealth literacy: the figure/model as it stands is a bit one dimensional. Suggest distinguishing the different levels as per Dahlgren and Whitehead and be more explicit about the left side that distinguishes determinants and outcomes.*

Reply:

Thank you for your suggestion. We have made some revisions to the figure to improve its clarity. Firstly, we have made a more explicit distinction between the different levels of the determinants and their interactions, as per Dahlgren and Whitehead's model. Secondly, we have separated the groups of determinants and outcomes into upper and lower parts using a bold line to make them more visible. We have also created dashed arrows to indicate non-causal relationships between the determinants and eHealth literacy, as well as between eHealth literacy and the outcomes. Furthermore, we have moved the group of determinants to the upper part of the figure, and the outcomes to the lower part. Lastly, we have included an explanation of the figure in the caption.

**Figure 2. Conceptual model of eHealth literacy determinants and outcomes**

The model suggests that eHealth literacy is shaped by a complex interaction of multi-layered determinants (i.e. non-modifiable factors, individual lifestyle, social and community networks, living and working conditions, and general socio-economic and cultural conditions). As a result, eHealth literacy is also associated with various outcomes.

*Other
11. Abstract: background should include ‘healthy adults’.*

Reply: Thank you for your reminder. We have revised the background section in our abstract accordingly.

*12. The following sentence appears to be incorrectly in the article: “Please add subheading to guide the classification of determinants according to the model” (p36).*

Reply: Thank you for your meticulous review. We have deleted that sentence.

*13. Discussion: need to draw out more the differences between different countries/parts of the world?*

Reply:

Thank you for your comment on our article. We appreciate your suggestion to draw out more the differences between different countries in the discussion section. We have compared the results of our SR with those from the previous systematic reviews focused on different countries in these two paragraphs:

(Discussion, page 41) …This factor includes education, employment, income and ease of paying for health care, all of which play important roles in determining the eHealth literacy levels of healthy adults. This finding is consistent with the result of a previous systematic review of eHealth literacy in an underserved population in the US [19]. In the countries where there is no universal health coverage, such as the US, those who are poor and uninsured may have lesser access to health care, confirming their status as a vulnerable and underserved population [19]. Economically disadvantaged people may also have decreased opportunity to access technology [19]….

(Discussion, page 42) A systematic review on the influencing factors of eHealth literacy among Chinese older adults [8] showed several similar findings with our study results, i.e. age, gender, education, socioeconomic status, and language influenced the levels of eHealth literacy [8]. These similarities suggest the factors which may be the universal determinants of eHealth literacy across different culture and population. However, our present study found that eHealth literacy was also influenced by literacy factors and ICT infrastructure. Another difference is that physical and psychological conditions, marital status, and being the family carer were found to be associated with the Chinese older adults’ levels of eHealth literacy [8]. These differences may indicate the factors that may be unique to older adult population, especially in China. With regards to outcomes, similar to our study findings, a recent systematic review concluded that eHealth literacy is associated with health-promoting behaviours including regular eating and exercise, and compliance with disease prevention behaviours [27]. These similar and different findings show the importance of conducting systematic review to find the consistent and inconsistent patterns across different studies.

*14. Some of the language in the article is awkward. Suggest reviewing for structure of sentences.*

Reply:

Thank you for your feedback. We have our article professionally proofread and edited by a native English academic and proofreader. We also have reviewed our article to improve its readability and clarity.
