# Supplementary material for: Determinants and outcomes of eHealth literacy in healthy adults: A systematic review
Source: PLoS One. 2023 Oct 4;18(10):e0291229. doi: 10.1371/journal.pone.0291229 (PMC10550189; doi:10.1371/journal.pone.0291229)
Supplement: Attachment — Submitted filename: response to reviewers_23Aug23.docx [file pone.0291229.s005.docx]

**PONE-D-22-35330R1 -** Determinants and outcomes of eHealth literacy in healthy adults: A systematic review

PLOS ONE Academic Editor

Dr. Muhammad Shahzad Aslam, Ph.D.,M.Phil., Pharm-D

Dear Dr Aslam,

On behalf of all authors, I would like to thank the academic editor and reviewers for the valuable feedback to improve the quality of our manuscript. The editor' comments are listed below in italicised font, while our responses are given in normal font. Changes or additions we made in the manuscripts are marked with yellow highlight and red color. The existing contents which address your comments are marked with purple color.

**List of response**

1-*It is necessary to provide a reproducible search strategy. The current given information does not determine the reproducibility. Please provide detail search strategy.*

Response:

Thank you for your comment. We have added a supporting information file containing our detailed search strategy for each databases that we used.

**Search strategy**

We searched six electronic databases: MEDLINE, EMBASE, PsycINFO, CINAHL, Cochrane Library and ProQuest to identify relevant studies. Relevant grey literature including dissertations and theses were identified from ProQuest. These databases were selected since they are among the largest and most widely recognized as authoritative sources in the fields of healthcare, medical, nursing, psychology, and behavioral sciences. They offer a comprehensive coverage of literature relevant to the eHealth literacy topic. Including other databases might introduce redundancy in the review process. These databases were chosen to strike a balance between comprehensive coverage and efficiency.

A search strategy was developed to capture the key elements of the topic of interest, i.e.: determinants and outcomes of eHealth literacy. Keywords were combined using AND and OR Boolean operators. The year of publication was not limited. The detailed search strategy is presented in appendix 1.

(page 4)

2- *how did you removing duplicates. The current methodology in incomplete. Please read https://jbi.global/. Please make sure your methodology is in details and include all aspect of data screening method, data collection methods.*

Response:

Thank you for your suggestion, We have checked and referred to JBI guidelines to add more details in our methods.

All citations were managed using EndNote 20 software (Clarivate Analytics, PA, USA). Duplicates were identified and removed electronically (using 'Find Duplicates' feature in EndNote) and manually. (page 4)

3-*Please provide us PICO. The PICO framework focuses on the Population, Intervention, Comparison and Outcomes. If you use other framework then give to us. It is mandatory.*

Response:

Thank you for your comments. We have written the population and outcomes in the eligibility criteria of our review. We also added that intervention and comparator were not within the scope of our review.

**Selection process**

Studies were included if they met these criteria: 1) studies examining ‘eHealth literacy’; 2) the type of study included observational and interventional studies, mixed methods studies, and qualitative studies; 3) population/participants were healthy adults (defined as individuals aged over 18-64 years [29] without significant chronic health conditions or disabilities); 4) the main outcomes were the determinants (i.e. influencing or associated factors) and outcomes (i.e. positive and negative outcomes) of eHealth literacy. Intervention and comparator were beyond the scope of this review. (page 4)

4-*Provide details on grey literature. Mandatory*

Response:

Thank you for your comment. We have added in our 'Search strategy' section that grey literature were searched from ProQuest.

**Search strategy**

We searched six electronic databases: MEDLINE, EMBASE, PsycINFO, CINAHL, Cochrane Library and ProQuest to identify relevant studies. Relevant grey literature including dissertations and theses were identified from ProQuest. These databases were selected since they are among the largest and most widely recognized as authoritative sources in the fields of healthcare, medical, nursing, psychology, and behavioral sciences. They offer a comprehensive coverage of literature relevant to the eHealth literacy topic. Including other databases might introduce redundancy in the review process. These databases were chosen to strike a balance between comprehensive coverage and efficiency.

A search strategy was developed to capture the key elements of the topic of interest, i.e.: determinants and outcomes of eHealth literacy. Keywords were combined using AND and OR Boolean operators. The year of publication was not limited. The detailed search strategy is presented in appendix 1.

(page 4)

5-*Provide justification on why you exclude other database. Mandatory*

Response:

We have explained our justification to include MEDLINE, EMBASE, PsycINFO, CINAHL, Cochrane Library and ProQuest and exclude other databases.

**Search strategy**

We searched six electronic databases: MEDLINE, EMBASE, PsycINFO, CINAHL, Cochrane Library and ProQuest to identify relevant studies. Relevant grey literature including dissertations and theses were identified from ProQuest. These databases were selected since they are among the largest and most widely recognized as authoritative sources in the fields of healthcare, medical, nursing, psychology, and behavioral sciences. They offer a comprehensive coverage of literature relevant to the eHealth literacy topic. Including other databases might introduce redundancy in the review process. These databases were chosen to strike a balance between comprehensive coverage and efficiency.

A search strategy was developed to capture the key elements of the topic of interest, i.e.: determinants and outcomes of eHealth literacy. Keywords were combined using AND and OR Boolean operators. The year of publication was not limited. The detailed search strategy is presented in appendix 1.

*5. a. Regarding Study risk of bias assessment. Kindly compare it with other methods such as Cochrane Risk of Bias Tool, Newcastle-Ottawa Scale (NOS), AXIS (Appraisal tool for Cross-Sectional Studies), AMSTAR 2 (A MeaSurement Tool to Assess systematic Reviews). The current information is not sufficient.*

Response:

Thank you for your suggestion. We have incorporated it in our paper.

**Assessment of methodological quality**

AM and AAP independently carried out the critical appraisal for each study. The Joanna Briggs Institute (JBI) critical appraisal tools were used, according to each study’s design, to assist in examining the trustworthiness and risk of bias of the included studies [30]. The JBI critical appraisal tools are widely recognized within the field of evidence synthesis for their comprehensive approach to evaluating the risk of bias based on the study’s design, conduct, and analysis. While other tools, such as the Cochrane Risk of Bias Tool, Newcastle-Ottawa Scale (NOS), Appraisal tool for Cross-Sectional Studies (AXIS), and A measurement Tool to Assess Systematic Reviews (AMSTAR 2), mostly focus on specific study types and their respective attributes, JBI tools offer a broader approach in comprehensively assessing various study designs. Therefore, the JBI tools were deemed suitable for systematically reviewing the determinants and outcomes of eHealth literacy across different research methodologies. (page 5)

6-*Kindly organize your table properly. All given tables has several structural flaws. Proof read properly.*

Response:

Thank you for your comments. We have rechecked the structure, and proofread the tables.

Additionally, table of characteristics of included studies has been moved from the main text to the Supporting information part. In that table, we removed the mean eHealth literacy score as it was deemed unnecessary.

7-*Provide heading and details, Study strength, study limitation, future direction. Mandatory.*

Response:

Thank you for your suggestion.

We have provided headings for the study strengths, limitations and future directions (the future directions was put after the Conclusions). In addition, we also incorporated the study limitations and future directions in the Discussion section to improve clarity and contextualisation (please see below examples).

Discussion, Paragraph 1, page 16:

(Limitation) However, this review did not include studies among older adults, therefore, comparisons between younger and older age participants cannot be made. In terms of sex, it is quite surprising that men were more likely to have a higher level of eHealth literacy than women. Prior studies showed that women used health apps [76] and searched health information online [77, 78] more often than men did. Women were also found to be more eHealth literate than men and perceived online information quality better [79, 80]. (Future direction) More studies are needed to clarify how sex differences determine eHealth literacy.

Paragraph 3, page 17:

(Future direction) Further studies are deemed necessary to update eHEALS to fit its purpose in the Web 2.0 internet era. (Future direction) Subscales to assess communication skills in online social interactions and in synthesising contents are some instances of items that could be developed for updated version of eHEALS. (Future direction) Studies are also needed to assess the traditional literacy, scientific literacy and media literacy. The latter is especially important in combating disinformation and bridging the digital divide [82].

Implications for policymaking (page 17-18)

Referring to Dahlgren and Whitehead’s model [31], different policy levels may apply in different layers of the health determinant. Policy level 1 is for the major structural environment, in which the language factor is situated. For addressing this determinant, national-level strategies, such as incorporating language services in the national health care system, are needed [83]. Having national and health industry investments to develop population-based language services by using ICT is also recommended [83]. Furthermore, policy level 2, which aims to improve living and working conditions can achieve that aim by, for example, a) providing the welfare benefits through the social security sector, and improving b) health care services, c) the education system and d) the ICT infrastructure [31]. Policy level 3, aiming at strengthening social and community support is not applicable, as there is no eHealth literacy determinant identified in this layer. Policy level 4 aims at influencing individual lifestyles [31]. The policy can include developing programmes to enhance eHealth literacy skills. Finally, for the innermost layer, i.e. the non-modifiable factor, the intervention can be integrated into the educational improvement of the eHealth literacy for older adults of both sexes and women of all ages.

**Study strengths**

The systematic review offers comprehensive summ

arized evidence of the determinants and outcomes of eHealth literacy in healthy adults. It encompasses a wide range of factors, both non-modifiable and individual, as well as environmental and cultural aspects. In addition, the review acknowledges that different layers of eHealth literacy determinants can have varying policy implications. This recognition is crucial for policymakers and healthcare professionals seeking to enhance eHealth literacy, as it provides insights into targeted interventions and strategies.

**Study limitations**

As with all systematic reviews, we cannot be fully certain that we have identified all relevant literature. This review only covered six databases and studies published in English. We may have missed potentially eligible studies published in other languages or contained within other databases. We only included articles which measured associations of eHealth literacy with the determinant or outcome variables. Articles presenting only differences between those variables were excluded. This constraint might also limit the findings of the review. Furthermore, we limit our population to healthy adults, who may not be the vulnerable population with regards to eHealth literacy. It is important to note that the studies included a relatively diverse group of healthy adults, e.g. students and healthcare professionals. While such inclusion can provide a more comprehensive picture of eHealth literacy in healthy adults, it can also introduce heterogeneity into the study population. Therefore, careful consideration of how the results is interpreted and generalized is needed.

**Conclusions and future directions (page 19 & 20)**

This systematic review has contributed to the understanding of the putative determinants and outcomes of eHealth literacy in healthy adults. In summary, the determinants of eHealth literacy include the non-modifiable factors (age, sex); individual factors (literacy, online health information seeking behavior); living and working conditions (education, employment, health care service and ICT infrastructure) and general cultural conditions (particularly language). Whereas, the outcomes of eHealth literacy include health promotion behaviors, COVID-19 preventive behaviors, psychological wellbeing, better social and community networks, better self-rated health and better health service utilisation. Different layers of eHealth literacy determinants can have different policy implications as they aim to improve eHealth literacy.

Future studies should incorporate artificial intelligence (AI) into the eHealth landscape. One possible direction is the expansion of the eHealth Literacy tool to include AI-driven health information. AI can also be used to develop inclusive eHealth solutions that are tailored to different literacy levels. The key to AI's influence is personalization, which enables the creation of eHealth intervention finely tuned to individual literacy levels. This personalisation encourages more effective health information dissemination while improving accessibility.

8-*Please revisit PRIMA flow diagram and cross-check the statement and content provided.*

Response:

Thank you for your suggestion.

We have revisited our PRISMA flow diagram and checked its content for accuracy. We have corrected the number of excluded articles - apologies for the mistake.
