# Supplementary material for: Determinants and outcomes of eHealth literacy in healthy adults: A systematic review
Source: PLoS One. 2023 Oct 4;18(10):e0291229. doi: 10.1371/journal.pone.0291229 (PMC10550189; doi:10.1371/journal.pone.0291229)
Supplement: Peer review history

# Peer review: Decision Letter 0

**DOI:** 10.1371/journal.pone.0291229.r001

**Authors:** Guglielmo Campus

14 Feb 2023

PONE-D-22-35330

Determinants and outcomes of eHealth literacy in healthy adults: A systematic review

PLOS ONE

Dear Dr. Milanti,

Thank you for submitting your manuscript to PLOS ONE. After careful consideration, we feel that it has merit but does not fully meet PLOS ONE’s publication criteria as it currently stands. Therefore, we invite you to submit a revised version of the manuscript that addresses the points raised during the review process.

Please submit your revised manuscript by Mar 31 2023 11:59PM. If you will need more time than this to complete your revisions, please reply to this message or contact the journal office at plosone@plos.org. When you're ready to submit your revision, log on to https://www.editorialmanager.com/pone/ and select the 'Submissions Needing Revision' folder to locate your manuscript file.

Please include the following items when submitting your revised manuscript:

- A rebuttal letter that responds to each point raised by the academic editor and reviewer(s). You should upload this letter as a separate file labeled 'Response to Reviewers'.
- A marked-up copy of your manuscript that highlights changes made to the original version. You should upload this as a separate file labeled 'Revised Manuscript with Track Changes'.
- An unmarked version of your revised paper without tracked changes. You should upload this as a separate file labeled 'Manuscript'.

If you would like to make changes to your financial disclosure, please include your updated statement in your cover letter. Guidelines for resubmitting your figure files are available below the reviewer comments at the end of this letter.

If applicable, we recommend that you deposit your laboratory protocols in protocols.io to enhance the reproducibility of your results. Protocols.io assigns your protocol its own identifier (DOI) so that it can be cited independently in the future. For instructions see: https://journals.plos.org/plosone/s/submission-guidelines#loc-laboratory-protocols. Additionally, PLOS ONE offers an option for publishing peer-reviewed Lab Protocol articles, which describe protocols hosted on protocols.io. Read more information on sharing protocols at https://plos.org/protocols?utm_medium=editorial-email&utm_source=authorletters&utm_campaign=protocols.

We look forward to receiving your revised manuscript.

Kind regards,

Guglielmo Campus, Ph.D DDS

Academic Editor

PLOS ONE

Journal Requirements:

When submitting your revision, we need you to address these additional requirements.

1. Please ensure that your manuscript meets PLOS ONE's style requirements, including those for file naming. The PLOS ONE style templates can be found at

https://journals.plos.org/plosone/s/file?id=wjVg/PLOSOne_formatting_sample_main_body.pdf and

https://journals.plos.org/plosone/s/file?id=ba62/PLOSOne_formatting_sample_title_authors_affiliations.pdf

2. In your Data Availability statement, you have not specified where the minimal data set underlying the results described in your manuscript can be found. PLOS defines a study's minimal data set as the underlying data used to reach the conclusions drawn in the manuscript and any additional data required to replicate the reported study findings in their entirety. All PLOS journals require that the minimal data set be made fully available. For more information about our data policy, please see http://journals.plos.org/plosone/s/data-availability.

Upon re-submitting your revised manuscript, please upload your study’s minimal underlying data set as either Supporting Information files or to a stable, public repository and include the relevant URLs, DOIs, or accession numbers within your revised cover letter. For a list of acceptable repositories, please see http://journals.plos.org/plosone/s/data-availability#loc-recommended-repositories. Any potentially identifying patient information must be fully anonymized.

Important: If there are ethical or legal restrictions to sharing your data publicly, please explain these restrictions in detail. Please see our guidelines for more information on what we consider unacceptable restrictions to publicly sharing data: http://journals.plos.org/plosone/s/data-availability#loc-unacceptable-data-access-restrictions. Note that it is not acceptable for the authors to be the sole named individuals responsible for ensuring data access.

We will update your Data Availability statement to reflect the information you provide in your cover letter.

3. We note you have included a table to which you do not refer in the text of your manuscript. Please ensure that you refer to Table 4 in your text; if accepted, production will need this reference to link the reader to the Table.

4. Please review your reference list to ensure that it is complete and correct. If you have cited papers that have been retracted, please include the rationale for doing so in the manuscript text, or remove these references and replace them with relevant current references. Any changes to the reference list should be mentioned in the rebuttal letter that accompanies your revised manuscript. If you need to cite a retracted article, indicate the article’s retracted status in the References list and also include a citation and full reference for the retraction notice.

[Note: HTML markup is below. Please do not edit.]

Reviewers' comments:

Reviewer's Responses to Questions

**Comments to the Author**

1. Is the manuscript technically sound, and do the data support the conclusions?

The manuscript must describe a technically sound piece of scientific research with data that supports the conclusions. Experiments must have been conducted rigorously, with appropriate controls, replication, and sample sizes. The conclusions must be drawn appropriately based on the data presented. 

Reviewer #1: Yes

Reviewer #2: Partly

**********

2. Has the statistical analysis been performed appropriately and rigorously? 

Reviewer #1: Yes

Reviewer #2: I Don't Know

**********

3. Have the authors made all data underlying the findings in their manuscript fully available?

The PLOS Data policy requires authors to make all data underlying the findings described in their manuscript fully available without restriction, with rare exception (please refer to the Data Availability Statement in the manuscript PDF file). The data should be provided as part of the manuscript or its supporting information, or deposited to a public repository. For example, in addition to summary statistics, the data points behind means, medians and variance measures should be available. If there are restrictions on publicly sharing data—e.g. participant privacy or use of data from a third party—those must be specified.

Reviewer #1: Yes

Reviewer #2: Yes

**********

4. Is the manuscript presented in an intelligible fashion and written in standard English?

PLOS ONE does not copyedit accepted manuscripts, so the language in submitted articles must be clear, correct, and unambiguous. Any typographical or grammatical errors should be corrected at revision, so please note any specific errors here.

Reviewer #1: Yes

Reviewer #2: Yes

**********

5. Review Comments to the Author

Please use the space provided to explain your answers to the questions above. You may also include additional comments for the author, including concerns about dual publication, research ethics, or publication ethics. (Please upload your review as an attachment if it exceeds 20,000 characters)

Reviewer #1: Thank you for the opportunity to read this paper. It address an important topic and contributes to the body of knowledge in this field. I only have few minor comments for authors' consideration.

Introduction

- Please elaborate why the authors want to understand eHealth literacy in healthy adults?

- Why it is important to understand determinants and outcomes?

Results

- It is worth noting that studies included a wide range of participants (e.g. carers or healthcare professionals), please comment on how it may affect the results of this SR.

Discussion

- I think readers may be interested to whether the results of this SR in healthy adults differ from those in other populations (e.g. older adults). Please consider adding few sentences to elaborate.

Reviewer #2: This is an interesting review which examines the determinants and outcomes of eHealth literacy.

Introduction/Background

- Rationale: given that there are already three reviews that examine interventions for health literacy, it would be important to be more specific about how this current review adds to the already extensive reviews in the area, in particular as you state that the results are ‘putative’ and other reviews have focused specifically on interventions and the outcomes of interventions.

- A recently published systematic review: https://pubmed.ncbi.nlm.nih.gov/36716080/

- Why focus on healthy adults only and how are healthy adults defined?

- Rationale for how ‘outcomes’ can be described as benefits and disadvantages.

Methods:

- What software was used to screen the articles?

- Where were the data extracted to?

- Data Synthesis: the Dahlgren and Whitehead model is explained, but not how the data were synthesised and applied to this model. How was this undertaken from a methods perspective?

- I don’t think Table 2 or Table 3 are necessary. These could be put in a supplementary file. Also, no need to list the JBI critical appraisal questions.

Results/Discussion

- I would question the appropriateness or need to use of the term ‘putative’ throughout the article. If there is a description of what is meant by putative and why these studies are putative determinants and outcomes only in the background (or early results) and perhaps again in the conclusion, there is no need to repeat this throughout the article.

- Conceptual model of ehealth literacy: the figure/model as it stands is a bit one dimensional. Suggest distinguishing the different levels as per Dahlgren and Whitehead and be more explicit about the left side that distinguishes determinants and outcomes.

Other

- Abstract: background should include ‘healthy adults’.

- The following sentence appears to be incorrectly in the article: “Please add subheading to guide the classification of determinants according to the model” (p36).

- Discussion: need to draw out more the differences between different countries/parts of the world?

- Some of the language in the article is awkward. Suggest reviewing for structure of sentences.

**********

6. PLOS authors have the option to publish the peer review history of their article (what does this mean?). If published, this will include your full peer review and any attached files.

If you choose “no”, your identity will remain anonymous but your review may still be made public.

**Do you want your identity to be public for this peer review?** For information about this choice, including consent withdrawal, please see our Privacy Policy.

Reviewer #1: No

Reviewer #2: No

**********​

[NOTE: If reviewer comments were submitted as an attachment file, they will be attached to this email and accessible via the submission site. Please log into your account, locate the manuscript record, and check for the action link "View Attachments". If this link does not appear, there are no attachment files.]

While revising your submission, please upload your figure files to the Preflight Analysis and Conversion Engine (PACE) digital diagnostic tool, https://pacev2.apexcovantage.com/. PACE helps ensure that figures meet PLOS requirements. To use PACE, you must first register as a user. Registration is free. Then, login and navigate to the UPLOAD tab, where you will find detailed instructions on how to use the tool. If you encounter any issues or have any questions when using PACE, please email PLOS at figures@plos.org. Please note that Supporting Information files do not need this step.

# Author comment: Author response to Decision Letter 0

**DOI:** 10.1371/journal.pone.0291229.r002

30 Apr 2023

Dear Editors and Reviewers: 

On behalf of all authors, I would like to thank the editor and all reviewers for the valuable feedback that we have used to improve the quality of our manuscript. The reviewer comments are listed below in italicised font, while our responses are given in normal font. Changes or additions we made in the manuscripts are marked with red colour. 

List of response

Reviewer #1: 

Thank you for the opportunity to read this paper. It address an important topic and contributes to the body of knowledge in this field. I only have few minor comments for authors' consideration.

Introduction

1. Please elaborate why the authors want to understand eHealth literacy in healthy adults?

Reply: 

Thank you for your valuable suggestion. We have taken your feedback into consideration and have elaborated on our rationale for studying eHealth literacy in healthy adults. We have included our response in the introduction section of our manuscript, specifically in the last paragraph.

Understanding the determinants and outcomes of eHealth literacy can provide valuable insights into the factors that influence individuals’ ability to use technology to access, understand, and use health information, and the impact that eHealth literacy has on health outcomes. This information can then be used to develop interventions and policies to promote eHealth literacy and improve health outcomes in healthy adults. Focusing on the adult population is important since this population makes up the largest age group in the world [28]. Moreover, by focusing the study population on healthy adults, the researchers can minimise the potential confounding effects of health conditions or other factors that may influence eHealth literacy. (page 2-3)

2. Why it is important to understand determinants and outcomes?

Reply: 

Thank you for your comment. We believe that understanding the determinants and outcomes of eHealth literacy is crucial because it can help identify the factors that contribute to individuals' ability to access and use health information through technology, and how this ability impacts their health outcomes. By examining these factors, we can develop interventions and policies that promote eHealth literacy and improve health outcomes in healthy adults. 

We have added this explanation to the last paragraph of our introduction section. 

Understanding the determinants and outcomes of eHealth literacy can provide valuable insights into the factors that influence individuals’ ability to use technology to access, understand, and use health information, and the impact that eHealth literacy has on health outcomes. This information can then be used to develop interventions and policies to promote eHealth literacy and improve health outcomes in healthy adults. Focusing on the adult population is important since this population makes up the largest age group in the world [28]. Moreover, by focusing the study population on healthy adults, the researchers can minimise the potential confounding effects of health conditions or other factors that may influence eHealth literacy. (page 2-3)

Results

3. It is worth noting that studies included a wide range of participants (e.g. carers or healthcare professionals), please comment on how it may affect the results of this SR.

Reply: 

Thank you for raising this issue. We agree that it is important to note about the wide range of participants which may influence the results of our SR. We have acknowledged it in the following comment at the end of the discussion section. 

It is important to note that the studies included a diverse kind of healthy adults, e.g. students and healthcare professionals. While such inclusion can provide a more comprehensive picture of eHealth literacy in healthy adults, it can also introduce heterogeneity into the study population. Therefore, careful consideration of how the results are interpreted and generalized is needed. (page 44)

Discussion

4. I think readers may be interested to whether the results of this SR in healthy adults differ from those in other populations (e.g. older adults). Please consider adding few sentences to elaborate.

Reply: 

Thank you for your suggestion. We have written an additional paragraph in the discussion section to elaborate the similarities and differences between our SR and the most relevant previous SR. 

A systematic review on the influencing factors of eHealth literacy among Chinese older adults [8] showed several similar findings with our study results, i.e. age, gender, education, socioeconomic status, and language influenced the levels of eHealth literacy [8]. These similarities suggest the factors which may be the universal determinants of eHealth literacy across different culture and population. However, our present study found that eHealth literacy was also influenced by literacy factors and ICT infrastructure. Another difference is that physical and psychological conditions, marital status, and being the family carer were found to be associated with the Chinese older adults’ levels of eHealth literacy [8]. These differences may indicate the factors that may be unique to older adult population, especially in China. With regards to outcomes, similar to our study findings, a recent systematic review concluded that eHealth literacy is associated with health-promoting behaviours including regular eating and exercise, and compliance with disease prevention behaviours [27]. These similar and different findings show the importance of conducting systematic review to find the consistent and inconsistent patterns across different studies. (page 43)

Reviewer #2: 

This is an interesting review which examines the determinants and outcomes of eHealth literacy.

Introduction/Background

1. Rationale: given that there are already three reviews that examine interventions for health literacy, it would be important to be more specific about how this current review adds to the already extensive reviews in the area, in particular as you state that the results are ‘putative’ and other reviews have focused specifically on interventions and the outcomes of interventions.

Reply: 

Thank you for your meaningful feedback on our manuscript. We agree that it is important to clarify how our review adds to the existing literature on eHealth literacy, given the extensive previous reviews particularly on eHealth literacy interventions for older adults. 

Our review takes a broader approach by examining the determinants and outcomes of eHealth literacy in healthy adults, while previous reviews on eHealth literacy interventions focused on older adults. Focusing on the adult population is important since this population makes up the largest age group in the world. Moreover, by focusing the study population on healthy adults, the researchers can minimise the potential confounding effects of health conditions or other factors that may influence eHealth literacy. In addition, our review offers a model of eHealth literacy determinants and outcomes which can be helpful to outline strategic approaches to promote greater equity in eHealth literacy through different policy levels. Finally, our present review found the factors unique to healthy adults which are not found among older adult population as well as the similar factors across these populations (as illustrated in the second last paragraph in the discussion section).

We also added an explanation about this rationale to the last paragraph of our introduction: 

“However, to the best of our knowledge, there is still no systematic review to summarise the evidence of eHealth literacy’s determinants and outcomes, unrelated to a specific intervention. To address this perceived gap, this systematic review aims to summarise and appraise the existing research which has examined the determinants and outcomes of eHealth literacy in healthy adults. Understanding the determinants and outcomes of eHealth literacy can provide valuable insights into the factors that influence individuals’ ability to use technology to access, understand, and use health information, and the impact that eHealth literacy has on health outcomes. This information can then be used to develop interventions and policies to promote eHealth literacy and improve health outcomes in healthy adults. Focusing on the adult population is important since this population makes up the largest age group in the world [28]. Moreover, by focusing the study population on healthy adults, the researchers can minimise the potential confounding effects of health conditions or other factors that may influence eHealth literacy.” (page 2-3)

2. A recently published systematic review: https://pubmed.ncbi.nlm.nih.gov/36716080/

Reply: 

Thank you for suggesting a recently published SR. We have incorporated it in our introduction (page 2) and discussion sections (page 43). 

3. Why focus on healthy adults only and how are healthy adults defined?

Reply: 

Thank you for your questions. We added these sentences to the end of our introduction section to answer your question:

“Focusing on the adult population is important since this population makes up the largest age group in the world [2]. Moreover, by focusing the study population on healthy adults, the researchers can minimise the potential confounding effects of health conditions or other factors that may influence eHealth literacy.” (page 3) 

Also, we have inserted the definition of healthy adults in the eligibility criteria, in the Methods section. 

Healthy adults are defined as individuals aged over 18-64 years (American Medical Association, 2022) without significant chronic health conditions or disabilities. (page 3)

4. Rationale for how ‘outcomes’ can be described as benefits and disadvantages.

Reply: 

Thank you for raising this issue. 

We replaced the use of terms ‘benefits and disadvantages’ with positive and negative outcomes (page 3). Describing outcomes as both positive and negative allows for a more balanced assessment of the eHealth literacy topic. 

Methods:

5. What software was used to screen the articles?

Reply: 

We used EndNote 20 software to screen the articles (we have added this information in our Methods section, page 3). 

6. Where were the data extracted to?

Reply: 

The data were extracted to an extraction sheet in Microsoft Word (we have added this information in our Methods section page 4). 

7. Data Synthesis: the Dahlgren and Whitehead model is explained, but not how the data were synthesised and applied to this model. How was this undertaken from a methods perspective?

Reply: 

Thank you for raising this issue. 

From a methods perspective, we synthesised the data from the included studies to this model by extracting the information on the determinants and outcomes of eHealth literacy and mapping the findings onto the model. Finally we identified areas where interventions or policies can be targeted related to each determinants. (We have incorporated these information at the end of the Data Synthesis part, Methods section) 

Data from the included studies were synthesised and applied to this model by extracting the information on the determinants and outcomes of eHealth literacy and mapping the findings onto the layers of Dahlgren and Whitehead’s model. Finally, areas where interventions or policies can be targeted related to each determinant were identified. (page 4) 

8. I don’t think Table 2 or Table 3 are necessary. These could be put in a supplementary file. Also, no need to list the JBI critical appraisal questions.

Reply: 

Thank you for your feedback. We have put table 2 and 3 in the Supplementary material. 

Results/Discussion

9. I would question the appropriateness or need to use of the term ‘putative’ throughout the article. If there is a description of what is meant by putative and why these studies are putative determinants and outcomes only in the background (or early results) and perhaps again in the conclusion, there is no need to repeat this throughout the article.

Reply: 

Thank you for your suggestion. We have removed the term ‘putative’, except in the early part of the Results section (As the causal relationship could not be drawn from the cross-sectional study design, the determinants and outcomes were putative only. Page 5) and in the Conclusions. 

10. Conceptual model of ehealth literacy: the figure/model as it stands is a bit one dimensional. Suggest distinguishing the different levels as per Dahlgren and Whitehead and be more explicit about the left side that distinguishes determinants and outcomes.

Reply: 

Thank you for your suggestion. We have made some revisions to the figure to improve its clarity. Firstly, we have made a more explicit distinction between the different levels of the determinants and their interactions, as per Dahlgren and Whitehead's model. Secondly, we have separated the groups of determinants and outcomes into upper and lower parts using a bold line to make them more visible. We have also created dashed arrows to indicate non-causal relationships between the determinants and eHealth literacy, as well as between eHealth literacy and the outcomes. Furthermore, we have moved the group of determinants to the upper part of the figure, and the outcomes to the lower part. Lastly, we have included an explanation of the figure in the caption.

Figure 2. Conceptual model of eHealth literacy determinants and outcomes

The model suggests that eHealth literacy is shaped by a complex interaction of multi-layered determinants (i.e. non-modifiable factors, individual lifestyle, social and community networks, living and working conditions, and general socio-economic and cultural conditions). As a result, eHealth literacy is also associated with various outcomes. 

Other

11. Abstract: background should include ‘healthy adults’.

Reply: Thank you for your reminder. We have revised the background section in our abstract accordingly. 

12. The following sentence appears to be incorrectly in the article: “Please add subheading to guide the classification of determinants according to the model” (p36).

Reply: Thank you for your meticulous review. We have deleted that sentence. 

13. Discussion: need to draw out more the differences between different countries/parts of the world?

Reply: 

Thank you for your comment on our article. We appreciate your suggestion to draw out more the differences between different countries in the discussion section. We have compared the results of our SR with those from the previous systematic reviews focused on different countries in these two paragraphs: 

(Discussion, page 41) …This factor includes education, employment, income and ease of paying for health care, all of which play important roles in determining the eHealth literacy levels of healthy adults. This finding is consistent with the result of a previous systematic review of eHealth literacy in an underserved population in the US [19]. In the countries where there is no universal health coverage, such as the US, those who are poor and uninsured may have lesser access to health care, confirming their status as a vulnerable and underserved population [19]. Economically disadvantaged people may also have decreased opportunity to access technology [19]….

(Discussion, page 42) A systematic review on the influencing factors of eHealth literacy among Chinese older adults [8] showed several similar findings with our study results, i.e. age, gender, education, socioeconomic status, and language influenced the levels of eHealth literacy [8]. These similarities suggest the factors which may be the universal determinants of eHealth literacy across different culture and population. However, our present study found that eHealth literacy was also influenced by literacy factors and ICT infrastructure. Another difference is that physical and psychological conditions, marital status, and being the family carer were found to be associated with the Chinese older adults’ levels of eHealth literacy [8]. These differences may indicate the factors that may be unique to older adult population, especially in China. With regards to outcomes, similar to our study findings, a recent systematic review concluded that eHealth literacy is associated with health-promoting behaviours including regular eating and exercise, and compliance with disease prevention behaviours [27]. These similar and different findings show the importance of conducting systematic review to find the consistent and inconsistent patterns across different studies.

14. Some of the language in the article is awkward. Suggest reviewing for structure of sentences.

Reply: 

Thank you for your feedback. We have our article professionally proofread and edited by a native English academic and proofreader. We also have reviewed our article to improve its readability and clarity.

Attachment Submitted filename: Response to reviewer_26042023.docx Click here for additional data file.

# Peer review: Decision Letter 1

**DOI:** 10.1371/journal.pone.0291229.r003

**Authors:** Muhammad Shahzad Aslam

7 Jul 2023

PONE-D-22-35330R1Determinants and outcomes of eHealth literacy in healthy adults: A systematic reviewPLOS ONE

Dear Dr. Milanti,

Thank you for submitting your manuscript to PLOS ONE. After careful consideration, we feel that it has merit but does not fully meet PLOS ONE’s publication criteria as it currently stands. Therefore, we invite you to submit a revised version of the manuscript that addresses the points raised during the review process.

1-It is necessary to provide a reproducible search strategy. The current given information does not determine the reproducibility. Please provide detail search strategy.

2- how did you removing duplicates. The current methodology in incomplete. Please read https://jbi.global/. Please make sure your methodology is in details and include all aspect of data screening method, data collection methods.

3-Please provide us PICO. The PICO framework focuses on the Population, Intervention, Comparison and Outcomes. If you use other framework then give to us. It is mandatory.

4-Provide details on grey literature. Mandatory

5-Provide justification on why you exclude other database. Mandatory

Regarding Study risk of bias assessment. Kindly compare it with other methods such as Cochrane Risk of Bias Tool, Newcastle-Ottawa Scale (NOS), AXIS (Appraisal tool for Cross-Sectional Studies), AMSTAR 2 (A MeaSurement Tool to Assess systematic Reviews). The current information is not sufficient.

6-Kindly organize your table properly. All given tables has several structural flaws. Proof read properly.

7-Provide heading and details, Study strength, study limitation, future direction. Mandatory.

8-Please revisit PRIMA flow dagram and cross-check the statement and content provided.

Please submit your revised manuscript by Aug 21 2023 11:59PM. If you will need more time than this to complete your revisions, please reply to this message or contact the journal office at plosone@plos.org. When you're ready to submit your revision, log on to https://www.editorialmanager.com/pone/ and select the 'Submissions Needing Revision' folder to locate your manuscript file.

Please include the following items when submitting your revised manuscript:

- A rebuttal letter that responds to each point raised by the academic editor and reviewer(s). You should upload this letter as a separate file labeled 'Response to Reviewers'.
- A marked-up copy of your manuscript that highlights changes made to the original version. You should upload this as a separate file labeled 'Revised Manuscript with Track Changes'.
- An unmarked version of your revised paper without tracked changes. You should upload this as a separate file labeled 'Manuscript'.

If you would like to make changes to your financial disclosure, please include your updated statement in your cover letter. Guidelines for resubmitting your figure files are available below the reviewer comments at the end of this letter.

If applicable, we recommend that you deposit your laboratory protocols in protocols.io to enhance the reproducibility of your results. Protocols.io assigns your protocol its own identifier (DOI) so that it can be cited independently in the future. For instructions see: https://journals.plos.org/plosone/s/submission-guidelines#loc-laboratory-protocols. Additionally, PLOS ONE offers an option for publishing peer-reviewed Lab Protocol articles, which describe protocols hosted on protocols.io. Read more information on sharing protocols at https://plos.org/protocols?utm_medium=editorial-email&utm_source=authorletters&utm_campaign=protocols.

We look forward to receiving your revised manuscript.

Kind regards,

Muhammad Shahzad Aslam, Ph.D.,M.Phil., Pharm-D

Academic Editor

PLOS ONE

Additional Editor Comments:

1-It is necessary to provide a reproducible search strategy. The current given information does not determine the reproducibility. Please provide detail search strategy.

2- how did you removing duplicates. The current methodology in incomplete. Please read https://jbi.global/. Please make sure your methodology is in details and include all aspect of data screening method, data collection methods.

3-Please provide us PICO. The PICO framework focuses on the Population, Intervention, Comparison and Outcomes. If you use other framework then give to us. It is mandatory.

4-Provide details on grey literature. Mandatory

5-Provide justification on why you exclude other database. Mandatory

Regarding Study risk of bias assessment. Kindly compare it with other methods such as Cochrane Risk of Bias Tool, Newcastle-Ottawa Scale (NOS), AXIS (Appraisal tool for Cross-Sectional Studies), AMSTAR 2 (A MeaSurement Tool to Assess systematic Reviews). The current information is not sufficient.

6-Kindly organize your table properly. All given tables has several structural flaws. Proof read properly.

7-Provide heading and details, Study strength, study limitation, future direction. Mandatory.

8-Please revisit PRIMA flow dagram and cross-check the statement and content provided.

[Note: HTML markup is below. Please do not edit.]

Reviewers' comments:

Reviewer's Responses to Questions

**Comments to the Author**

1. If the authors have adequately addressed your comments raised in a previous round of review and you feel that this manuscript is now acceptable for publication, you may indicate that here to bypass the “Comments to the Author” section, enter your conflict of interest statement in the “Confidential to Editor” section, and submit your "Accept" recommendation.

Reviewer #2: All comments have been addressed

**********

2. Is the manuscript technically sound, and do the data support the conclusions?

The manuscript must describe a technically sound piece of scientific research with data that supports the conclusions. Experiments must have been conducted rigorously, with appropriate controls, replication, and sample sizes. The conclusions must be drawn appropriately based on the data presented. 

Reviewer #2: Yes

**********

3. Has the statistical analysis been performed appropriately and rigorously? 

Reviewer #2: Yes

**********

4. Have the authors made all data underlying the findings in their manuscript fully available?

The PLOS Data policy requires authors to make all data underlying the findings described in their manuscript fully available without restriction, with rare exception (please refer to the Data Availability Statement in the manuscript PDF file). The data should be provided as part of the manuscript or its supporting information, or deposited to a public repository. For example, in addition to summary statistics, the data points behind means, medians and variance measures should be available. If there are restrictions on publicly sharing data—e.g. participant privacy or use of data from a third party—those must be specified.

Reviewer #2: Yes

**********

5. Is the manuscript presented in an intelligible fashion and written in standard English?

PLOS ONE does not copyedit accepted manuscripts, so the language in submitted articles must be clear, correct, and unambiguous. Any typographical or grammatical errors should be corrected at revision, so please note any specific errors here.

Reviewer #2: Yes

**********

6. Review Comments to the Author

Please use the space provided to explain your answers to the questions above. You may also include additional comments for the author, including concerns about dual publication, research ethics, or publication ethics. (Please upload your review as an attachment if it exceeds 20,000 characters)

Reviewer #2: (No Response)

**********

7. PLOS authors have the option to publish the peer review history of their article (what does this mean?). If published, this will include your full peer review and any attached files.

If you choose “no”, your identity will remain anonymous but your review may still be made public.

**Do you want your identity to be public for this peer review?** For information about this choice, including consent withdrawal, please see our Privacy Policy.

Reviewer #2: No

**********

[NOTE: If reviewer comments were submitted as an attachment file, they will be attached to this email and accessible via the submission site. Please log into your account, locate the manuscript record, and check for the action link "View Attachments". If this link does not appear, there are no attachment files.]

While revising your submission, please upload your figure files to the Preflight Analysis and Conversion Engine (PACE) digital diagnostic tool, https://pacev2.apexcovantage.com/. PACE helps ensure that figures meet PLOS requirements. To use PACE, you must first register as a user. Registration is free. Then, login and navigate to the UPLOAD tab, where you will find detailed instructions on how to use the tool. If you encounter any issues or have any questions when using PACE, please email PLOS at figures@plos.org. Please note that Supporting Information files do not need this step.

# Author comment: Author response to Decision Letter 1

**DOI:** 10.1371/journal.pone.0291229.r004

23 Aug 2023

PONE-D-22-35330R1 - Determinants and outcomes of eHealth literacy in healthy adults: A systematic review

PLOS ONE Academic Editor

Dr. Muhammad Shahzad Aslam, Ph.D.,M.Phil., Pharm-D

Dear Dr Aslam, 

On behalf of all authors, I would like to thank the academic editor and reviewers for the valuable feedback to improve the quality of our manuscript. The editor' comments are listed below in italicised font, while our responses are given in normal font. Changes or additions we made in the manuscripts are marked with yellow highlight and red color. The existing contents which address your comments are marked with purple color. 

List of response

1-It is necessary to provide a reproducible search strategy. The current given information does not determine the reproducibility. Please provide detail search strategy.

Response: 

Thank you for your comment. We have added a supporting information file containing our detailed search strategy for each databases that we used. 

Search strategy 

 We searched six electronic databases: MEDLINE, EMBASE, PsycINFO, CINAHL, Cochrane Library and ProQuest to identify relevant studies. Relevant grey literature including dissertations and theses were identified from ProQuest. These databases were selected since they are among the largest and most widely recognized as authoritative sources in the fields of healthcare, medical, nursing, psychology, and behavioral sciences. They offer a comprehensive coverage of literature relevant to the eHealth literacy topic. Including other databases might introduce redundancy in the review process. These databases were chosen to strike a balance between comprehensive coverage and efficiency. 

 A search strategy was developed to capture the key elements of the topic of interest, i.e.: determinants and outcomes of eHealth literacy. Keywords were combined using AND and OR Boolean operators. The year of publication was not limited. The detailed search strategy is presented in appendix 1. 

 (page 4)

2- how did you removing duplicates. The current methodology in incomplete. Please read https://jbi.global/. Please make sure your methodology is in details and include all aspect of data screening method, data collection methods.

Response: 

Thank you for your suggestion, We have checked and referred to JBI guidelines to add more details in our methods. 

All citations were managed using EndNote 20 software (Clarivate Analytics, PA, USA). Duplicates were identified and removed electronically (using 'Find Duplicates' feature in EndNote) and manually. (page 4) 

3-Please provide us PICO. The PICO framework focuses on the Population, Intervention, Comparison and Outcomes. If you use other framework then give to us. It is mandatory.

Response: 

Thank you for your comments. We have written the population and outcomes in the eligibility criteria of our review. We also added that intervention and comparator were not within the scope of our review.

Selection process 

 Studies were included if they met these criteria: 1) studies examining ‘eHealth literacy’; 2) the type of study included observational and interventional studies, mixed methods studies, and qualitative studies; 3) population/participants were healthy adults (defined as individuals aged over 18-64 years [29] without significant chronic health conditions or disabilities); 4) the main outcomes were the determinants (i.e. influencing or associated factors) and outcomes (i.e. positive and negative outcomes) of eHealth literacy. Intervention and comparator were beyond the scope of this review. (page 4)

4-Provide details on grey literature. Mandatory

Response: 

Thank you for your comment. We have added in our 'Search strategy' section that grey literature were searched from ProQuest. 

Search strategy 

 We searched six electronic databases: MEDLINE, EMBASE, PsycINFO, CINAHL, Cochrane Library and ProQuest to identify relevant studies. Relevant grey literature including dissertations and theses were identified from ProQuest. These databases were selected since they are among the largest and most widely recognized as authoritative sources in the fields of healthcare, medical, nursing, psychology, and behavioral sciences. They offer a comprehensive coverage of literature relevant to the eHealth literacy topic. Including other databases might introduce redundancy in the review process. These databases were chosen to strike a balance between comprehensive coverage and efficiency. 

 A search strategy was developed to capture the key elements of the topic of interest, i.e.: determinants and outcomes of eHealth literacy. Keywords were combined using AND and OR Boolean operators. The year of publication was not limited. The detailed search strategy is presented in appendix 1. 

(page 4)

5-Provide justification on why you exclude other database. Mandatory

Response: 

We have explained our justification to include MEDLINE, EMBASE, PsycINFO, CINAHL, Cochrane Library and ProQuest and exclude other databases. 

Search strategy 

 We searched six electronic databases: MEDLINE, EMBASE, PsycINFO, CINAHL, Cochrane Library and ProQuest to identify relevant studies. Relevant grey literature including dissertations and theses were identified from ProQuest. These databases were selected since they are among the largest and most widely recognized as authoritative sources in the fields of healthcare, medical, nursing, psychology, and behavioral sciences. They offer a comprehensive coverage of literature relevant to the eHealth literacy topic. Including other databases might introduce redundancy in the review process. These databases were chosen to strike a balance between comprehensive coverage and efficiency. 

 A search strategy was developed to capture the key elements of the topic of interest, i.e.: determinants and outcomes of eHealth literacy. Keywords were combined using AND and OR Boolean operators. The year of publication was not limited. The detailed search strategy is presented in appendix 1. 

5. a. Regarding Study risk of bias assessment. Kindly compare it with other methods such as Cochrane Risk of Bias Tool, Newcastle-Ottawa Scale (NOS), AXIS (Appraisal tool for Cross-Sectional Studies), AMSTAR 2 (A MeaSurement Tool to Assess systematic Reviews). The current information is not sufficient.

Response:

Thank you for your suggestion. We have incorporated it in our paper. 

Assessment of methodological quality 

AM and AAP independently carried out the critical appraisal for each study. The Joanna Briggs Institute (JBI) critical appraisal tools were used, according to each study’s design, to assist in examining the trustworthiness and risk of bias of the included studies [30]. The JBI critical appraisal tools are widely recognized within the field of evidence synthesis for their comprehensive approach to evaluating the risk of bias based on the study’s design, conduct, and analysis. While other tools, such as the Cochrane Risk of Bias Tool, Newcastle-Ottawa Scale (NOS), Appraisal tool for Cross-Sectional Studies (AXIS), and A measurement Tool to Assess Systematic Reviews (AMSTAR 2), mostly focus on specific study types and their respective attributes, JBI tools offer a broader approach in comprehensively assessing various study designs. Therefore, the JBI tools were deemed suitable for systematically reviewing the determinants and outcomes of eHealth literacy across different research methodologies. (page 5)

6-Kindly organize your table properly. All given tables has several structural flaws. Proof read properly.

Response: 

Thank you for your comments. We have rechecked the structure, and proofread the tables. 

Additionally, table of characteristics of included studies has been moved from the main text to the Supporting information part. In that table, we removed the mean eHealth literacy score as it was deemed unnecessary. 

7-Provide heading and details, Study strength, study limitation, future direction. Mandatory.

Response:

Thank you for your suggestion. 

We have provided headings for the study strengths, limitations and future directions (the future directions was put after the Conclusions). In addition, we also incorporated the study limitations and future directions in the Discussion section to improve clarity and contextualisation (please see below examples).

Discussion, Paragraph 1, page 16: 

(Limitation) However, this review did not include studies among older adults, therefore, comparisons between younger and older age participants cannot be made. In terms of sex, it is quite surprising that men were more likely to have a higher level of eHealth literacy than women. Prior studies showed that women used health apps [76] and searched health information online [77, 78] more often than men did. Women were also found to be more eHealth literate than men and perceived online information quality better [79, 80]. (Future direction) More studies are needed to clarify how sex differences determine eHealth literacy.

Paragraph 3, page 17: 

(Future direction) Further studies are deemed necessary to update eHEALS to fit its purpose in the Web 2.0 internet era. (Future direction) Subscales to assess communication skills in online social interactions and in synthesising contents are some instances of items that could be developed for updated version of eHEALS. (Future direction) Studies are also needed to assess the traditional literacy, scientific literacy and media literacy. The latter is especially important in combating disinformation and bridging the digital divide [82]. 

Implications for policymaking (page 17-18)

Referring to Dahlgren and Whitehead’s model [31], different policy levels may apply in different layers of the health determinant. Policy level 1 is for the major structural environment, in which the language factor is situated. For addressing this determinant, national-level strategies, such as incorporating language services in the national health care system, are needed [83]. Having national and health industry investments to develop population-based language services by using ICT is also recommended [83]. Furthermore, policy level 2, which aims to improve living and working conditions can achieve that aim by, for example, a) providing the welfare benefits through the social security sector, and improving b) health care services, c) the education system and d) the ICT infrastructure [31]. Policy level 3, aiming at strengthening social and community support is not applicable, as there is no eHealth literacy determinant identified in this layer. Policy level 4 aims at influencing individual lifestyles [31]. The policy can include developing programmes to enhance eHealth literacy skills. Finally, for the innermost layer, i.e. the non-modifiable factor, the intervention can be integrated into the educational improvement of the eHealth literacy for older adults of both sexes and women of all ages. 

Study strengths

The systematic review offers comprehensive summ

arized evidence of the determinants and outcomes of eHealth literacy in healthy adults. It encompasses a wide range of factors, both non-modifiable and individual, as well as environmental and cultural aspects. In addition, the review acknowledges that different layers of eHealth literacy determinants can have varying policy implications. This recognition is crucial for policymakers and healthcare professionals seeking to enhance eHealth literacy, as it provides insights into targeted interventions and strategies.

Study limitations

As with all systematic reviews, we cannot be fully certain that we have identified all relevant literature. This review only covered six databases and studies published in English. We may have missed potentially eligible studies published in other languages or contained within other databases. We only included articles which measured associations of eHealth literacy with the determinant or outcome variables. Articles presenting only differences between those variables were excluded. This constraint might also limit the findings of the review. Furthermore, we limit our population to healthy adults, who may not be the vulnerable population with regards to eHealth literacy. It is important to note that the studies included a relatively diverse group of healthy adults, e.g. students and healthcare professionals. While such inclusion can provide a more comprehensive picture of eHealth literacy in healthy adults, it can also introduce heterogeneity into the study population. Therefore, careful consideration of how the results is interpreted and generalized is needed. 

Conclusions and future directions (page 19 & 20)

 This systematic review has contributed to the understanding of the putative determinants and outcomes of eHealth literacy in healthy adults. In summary, the determinants of eHealth literacy include the non-modifiable factors (age, sex); individual factors (literacy, online health information seeking behavior); living and working conditions (education, employment, health care service and ICT infrastructure) and general cultural conditions (particularly language). Whereas, the outcomes of eHealth literacy include health promotion behaviors, COVID-19 preventive behaviors, psychological wellbeing, better social and community networks, better self-rated health and better health service utilisation. Different layers of eHealth literacy determinants can have different policy implications as they aim to improve eHealth literacy. 

 Future studies should incorporate artificial intelligence (AI) into the eHealth landscape. One possible direction is the expansion of the eHealth Literacy tool to include AI-driven health information. AI can also be used to develop inclusive eHealth solutions that are tailored to different literacy levels. The key to AI's influence is personalization, which enables the creation of eHealth intervention finely tuned to individual literacy levels. This personalisation encourages more effective health information dissemination while improving accessibility.

8-Please revisit PRIMA flow diagram and cross-check the statement and content provided.

Response: 

Thank you for your suggestion. 

We have revisited our PRISMA flow diagram and checked its content for accuracy.

Attachment Submitted filename: response to reviewers_23Aug23.docx Click here for additional data file.

# Peer review: Decision Letter 2

**DOI:** 10.1371/journal.pone.0291229.r005

**Authors:** Muhammad Shahzad Aslam

25 Aug 2023

Determinants and outcomes of eHealth literacy in healthy adults: A systematic review

PONE-D-22-35330R2

Dear Dr. Milanti,

We’re pleased to inform you that your manuscript has been judged scientifically suitable for publication and will be formally accepted for publication once it meets all outstanding technical requirements.

Within one week, you’ll receive an e-mail detailing the required amendments. When these have been addressed, you’ll receive a formal acceptance letter and your manuscript will be scheduled for publication.

An invoice for payment will follow shortly after the formal acceptance. To ensure an efficient process, please log into Editorial Manager at http://www.editorialmanager.com/pone/, click the 'Update My Information' link at the top of the page, and double check that your user information is up-to-date. If you have any billing related questions, please contact our Author Billing department directly at authorbilling@plos.org.

If your institution or institutions have a press office, please notify them about your upcoming paper to help maximize its impact. If they’ll be preparing press materials, please inform our press team as soon as possible -- no later than 48 hours after receiving the formal acceptance. Your manuscript will remain under strict press embargo until 2 pm Eastern Time on the date of publication. For more information, please contact onepress@plos.org.

Kind regards,

Muhammad Shahzad Aslam, Ph.D.,M.Phil., Pharm-D

Academic Editor

PLOS ONE

Additional Editor Comments (optional):

Reviewers' comments:

Reviewer's Responses to Questions

**Comments to the Author**

1. If the authors have adequately addressed your comments raised in a previous round of review and you feel that this manuscript is now acceptable for publication, you may indicate that here to bypass the “Comments to the Author” section, enter your conflict of interest statement in the “Confidential to Editor” section, and submit your "Accept" recommendation.

Reviewer #1: All comments have been addressed

Reviewer #2: All comments have been addressed

**********

2. Is the manuscript technically sound, and do the data support the conclusions?

The manuscript must describe a technically sound piece of scientific research with data that supports the conclusions. Experiments must have been conducted rigorously, with appropriate controls, replication, and sample sizes. The conclusions must be drawn appropriately based on the data presented. 

Reviewer #1: Yes

Reviewer #2: Yes

**********

3. Has the statistical analysis been performed appropriately and rigorously? 

Reviewer #1: Yes

Reviewer #2: N/A

**********

4. Have the authors made all data underlying the findings in their manuscript fully available?

The PLOS Data policy requires authors to make all data underlying the findings described in their manuscript fully available without restriction, with rare exception (please refer to the Data Availability Statement in the manuscript PDF file). The data should be provided as part of the manuscript or its supporting information, or deposited to a public repository. For example, in addition to summary statistics, the data points behind means, medians and variance measures should be available. If there are restrictions on publicly sharing data—e.g. participant privacy or use of data from a third party—those must be specified.

Reviewer #1: Yes

Reviewer #2: Yes

**********

5. Is the manuscript presented in an intelligible fashion and written in standard English?

PLOS ONE does not copyedit accepted manuscripts, so the language in submitted articles must be clear, correct, and unambiguous. Any typographical or grammatical errors should be corrected at revision, so please note any specific errors here.

Reviewer #1: Yes

Reviewer #2: Yes

**********

6. Review Comments to the Author

Please use the space provided to explain your answers to the questions above. You may also include additional comments for the author, including concerns about dual publication, research ethics, or publication ethics. (Please upload your review as an attachment if it exceeds 20,000 characters)

Reviewer #1: Thanks for the opportunity to review this paper. This systematic review addressed an important topic and filled the gaps in literature.

The author has responded to all my suggestions.

Reviewer #2: (No Response)

**********

7. PLOS authors have the option to publish the peer review history of their article (what does this mean?). If published, this will include your full peer review and any attached files.

If you choose “no”, your identity will remain anonymous but your review may still be made public.

**Do you want your identity to be public for this peer review?** For information about this choice, including consent withdrawal, please see our Privacy Policy.

Reviewer #1: No

Reviewer #2: **Yes: **Aisling Walsh

**********

# Editor report: Acceptance letter

**DOI:** 10.1371/journal.pone.0291229.r006

**Authors:** Muhammad Shahzad Aslam

25 Sep 2023

PONE-D-22-35330R2 

 Determinants and outcomes of eHealth literacy in healthy adults: A systematic review 

Dear Dr. Milanti:

I'm pleased to inform you that your manuscript has been deemed suitable for publication in PLOS ONE. Congratulations! Your manuscript is now with our production department. 

If your institution or institutions have a press office, please let them know about your upcoming paper now to help maximize its impact. If they'll be preparing press materials, please inform our press team within the next 48 hours. Your manuscript will remain under strict press embargo until 2 pm Eastern Time on the date of publication. For more information please contact onepress@plos.org.

If we can help with anything else, please email us at plosone@plos.org. 

Thank you for submitting your work to PLOS ONE and supporting open access. 

Kind regards, 

PLOS ONE Editorial Office Staff

on behalf of

Dr. Muhammad Shahzad Aslam 

Academic Editor

PLOS ONE
